# Long-term *in vitro* maintenance of clonal abundance and leukaemia-initiating potential in acute lymphoblastic leukaemia

**DOI:** 10.1038/leu.2016.79

**Published:** 2016-07-10

**Authors:** D Pal, H J Blair, A Elder, K Dormon, K J Rennie, D J L Coleman, J Weiland, K S Rankin, A Filby, O Heidenreich, J Vormoor

**Affiliations:** 1 Newcastle Cancer Centre at the Northern Institute for Cancer Research, Newcastle University, Newcastle Upon Tyne, UK; 2 Musculoskeletal Research Group, Institute of Cellular Medicine, Newcastle University, Newcastle Upon Tyne, UK; 3 Department of Hematology, Oncology, Hemostaseology, and Stem Cell Transplantation, Faculty of Medicine, RWTH Aachen University, Aachen, Germany; 4 Flow Cytometry Core Facility, Faculty of Medical Sciences, Newcastle University, Newcastle Upon Tyne, UK; 5 Great North Children's Hospital, Newcastle Upon Tyne Hospitals NHS Foundation Trust, Newcastle Upon Tyne, UK

## Abstract

Lack of suitable *in vitro* culture conditions for primary acute lymphoblastic leukaemia (ALL) cells severely impairs their experimental accessibility and the testing of new drugs on cell material reflecting clonal heterogeneity in patients. We show that Nestin-positive human mesenchymal stem cells (MSCs) support expansion of a range of biologically and clinically distinct patient-derived ALL samples. Adherent ALL cells showed an increased accumulation in the S phase of the cell cycle and diminished apoptosis when compared with cells in the suspension fraction. Moreover, surface expression of adhesion molecules CD34, CDH2 and CD10 increased several fold. Approximately 20% of the ALL cells were in G0 phase of the cell cycle, suggesting that MSCs may support quiescent ALL cells. Cellular barcoding demonstrated long-term preservation of clonal abundance. Expansion of ALL cells for >3 months compromised neither feeder dependence nor cancer initiating ability as judged by their engraftment potential in immunocompromised mice. Finally, we demonstrate the suitability of this co-culture approach for the investigation of drug combinations with luciferase-expressing primograft ALL cells. Taken together, we have developed a preclinical platform with patient-derived material that will facilitate the development of clinically effective combination therapies for ALL.

## Introduction

Robust preclinical models for childhood acute lymphoblastic leukaemia (ALL) are essential for dissecting mechanisms that drive malignant growth and survival and to test and develop novel targeted therapies that may improve current therapies with regard to efficacy and toxicity. Cell line models have been widely used in functional *in vitro* studies and preclinical drug screens.^1, 2, 3, 4, 5, 6^ Although cell lines do retain the original driver mutations, they do not represent the molecular complexity of the disease at presentation. More importantly, cell lines have adapted to suspension culture and grow without niche support. The combination of low complexity and reduced dependence on cell-extrinsic signalling can affect the translation of cell line data to the clinical situation, for example, in relation to clinically relevant mechanisms of drug resistance; ^7, 8, 9^ thus affecting the ability of cell line models to reflect the original disease.

Functional studies with primary blasts from children with ALL, however, have been hampered by the difficulty in expanding ALL cells *in vitro*. ALL blasts are highly dependent on their *in vivo* environment and rapidly undergo apoptosis *ex vivo*.^10, 11, 12^ Short-term *in vitro* assays have been developed for testing drug sensitivity;^13^ however, their use has not been widely implemented because of the rapid decline of ALL cells in these assays, even without exposure to any anti-leukaemic compounds.

The vast majority of ALL will engraft at low cell numbers and proliferate in highly immunodeficient mice.^14, 15, 16^ These studies have demonstrated that the murine bone marrow and lymphoid microenvironment is highly conserved between mice and men and able to support malignant human lymphoid cells. Although there is some evidence of clonal selection in the mice,^17, 18, 19, 20^ clonal complexity and niche dependency are preserved.^18^ Xenograft mouse models have been used for a wide range of studies including the phenotypic definition and homing of leukaemia propagating cells and for preclinical drug testing.^6, 16, 21, 22, 23, 24^ However, animal experiments are labour intensive, expensive and time consuming, thus limiting their application.

The group of Campana^11, 25^ has pioneered novel culture systems providing stroma support for the leukaemia blasts using immortalised mesenchymal stem cells (MSCs). These and similar bone marrow stromal co-cultures have been successfully used to model leukaemia–stroma adhesions and interactions.^26, 27, 28, 29, 30^ Stroma cells provide crucial survival signals to the leukaemic cells that affect drug resistance, thus mimicking the situation in patients.^10, 31, 32, 33, 34^ However, despite the ability of allogeneic bone marrow stromal cells to support proliferation of primary leukaemia blasts, acquisition of additional mutations following long-term cell expansion has been observed. This reduces the complexity and feeder dependence of the cultivated blasts, sometimes leading to the outgrowth of cell lines.^27, 28, 35^

We describe an optimised primary MSC culture system for the long-term propagation of a cytogenetically and clinically distinct panel of primary and primograft ALL blasts *in vitro* without compromising sample clonal composition and self-renewal capacity. We confirm early-passage primary MSCs to provide superior support than their counterpart feeders in B-ALL expansion. Combining MSC co-culture with lentiviral luciferase transduction of ALL cells generates an experimentally accessible medium–high throughput *in vitro* system that is suitable for preclinical drug testing in patient-derived primary leukaemic cells.

## Materials and methods

### Drug combination screen

This assay was carried on white frame and clear flat-bottom 96-well plates (Santa Cruz Biotechnology, Heidelberg, Germany) seeded with 10 000 MSCs per well. L707 cells were lentivirally transduced with pHR-cppt-SLIEW for stable expression of enhanced green fluorescent protein and firefly luciferase followed by sorting for green fluorescent protein positivity on a FACS Aria III (BD Biosciences, Oxford, UK) and expansion on MSCs for 2 weeks.^36^ For drug treatment, green fluorescent protein-positive ALL cells were seeded onto the 96-well plates at 5000 cells per well followed by incubation with clinically relevant drug concentrations. Dose response was analysed 5 days later by adding luciferin at 30 μg/ml (Promega, Southampton, UK) and measuring bioluminescence using the FLUOstar Omega microplate reader (BMG, Ortenberg, Germany). Combination indices were calculated for drug combinations in constant ratio to each other using software CalcuSyn Version 2.1.^37^

## Results

### MSCs support short-term proliferation of a range of clinically distinct patient-derived ALL cells

To compare the impact of stromal cell lines and primary human MSCs on B-ALL propagation, we examined ALL cell proliferation on either the murine stromal cell line M2-10B4, human TERT-immortalised MSC or primary human MSCs obtained from hip surgery.

Primary MSCs were characterised as NES+ CD73+ CDH2+ CD90++ TEK+ (expression of all these markers was homogenous across all MSCs); absence of haematopoietic cells was confirmed by lack of CD45 and CD19 surface expression (Supplementary Figure S3). We examined the growth of the t(12;21)-positive B-lineage ALL line REH on the feeder systems at very low cell concentration and low fetal calf serum condition. Under these conditions, REH cell numbers dropped ninefold within a week. In contrast, co-incubation with either M2-10B4 or MSCs resulted in a 5-fold and 15-fold increase in cell number, respectively, suggesting a superior growth-promoting role for human MSCs compared with the murine stroma cell line (Supplementary Figure S4).

**Figure 1:**
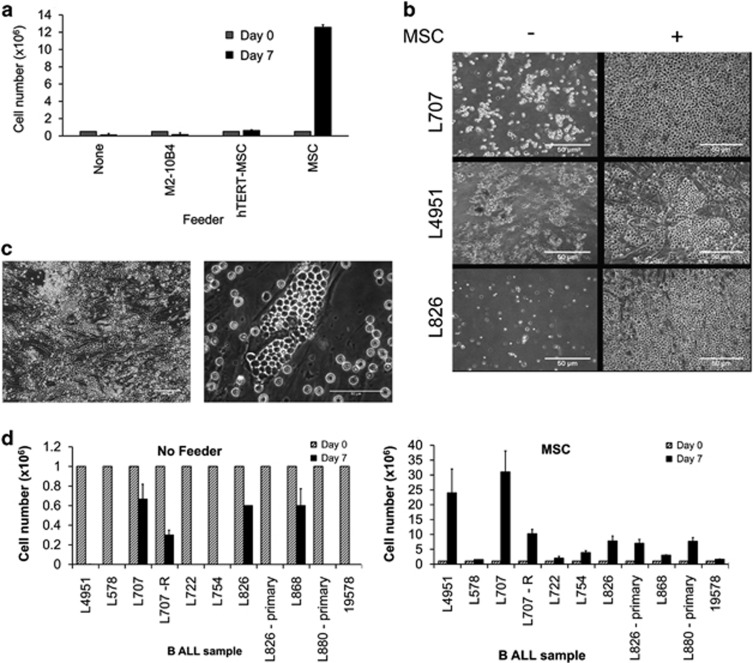
MSCs support ALL propagation *in vitro*. (**a**) Proliferation of L4951 cells without feeder, with murine M2-10B4, with TERT-MSC and with human MSC feeders. (**b**) Phase-contrast photographs of patient-derived B-ALL cells in suspension monocultures and in co-culture with MSCs. (**c**) Formation of cobblestone colonies by ALL cells on MSCs. (**d**) Proliferation of a panel of B-ALLs over 7 days without and with MSC feeder. For karyotype information see Supplementary Table S1. *N*⩾3 for all experiments shown; error bars, s.d.

Next, we studied the *ex vivo* expansion of ALL primograft material on these feeders. Replacement of RPMI-1640 by serum-free expansion medium and 20% fetal calf serum resulted in superior survival of primograft material (Supplementary Figure S5). Nevertheless, a t(9;22)-positive ALL primograft from patient L4951 showed poor viability and a more than fivefold reduced cell number in suspension culture that was only marginally improved by co-incubation with M2-10B4 cells (Figure 1a). However, co-culture on TERT-immortalised MSCs confirmed improved viability and successful maintenance of cell numbers, whereas incubation on an early-passage primary human MSC layer not only preserved primograft viability, but also induced formation of cobblestone pattern and 24-fold increased cell numbers indicating more than 4 cell doublings over 7 days (Figures 1a–c and Supplementary Figure S6). Furthermore, consistent with other existing reports,^38^ we confirm reduced Nestin expression in the TERT-MSC cells (Supplementary Figure S6).

We extended these cultures to a panel of nine different B-ALL primografts and two primary B-ALL samples (L880, L826) that had not been previously passaged through a mouse (Supplementary Table S1). Whereas all ALLs showed reduced cell numbers within 1 week of liquid culture, co-incubation with MSCs increased cell numbers by 1.5–30-fold (Figure 1d).

### Cell adherence instructs cell-cycle status and phenotype of the leukaemic blasts

**Figure 2:**
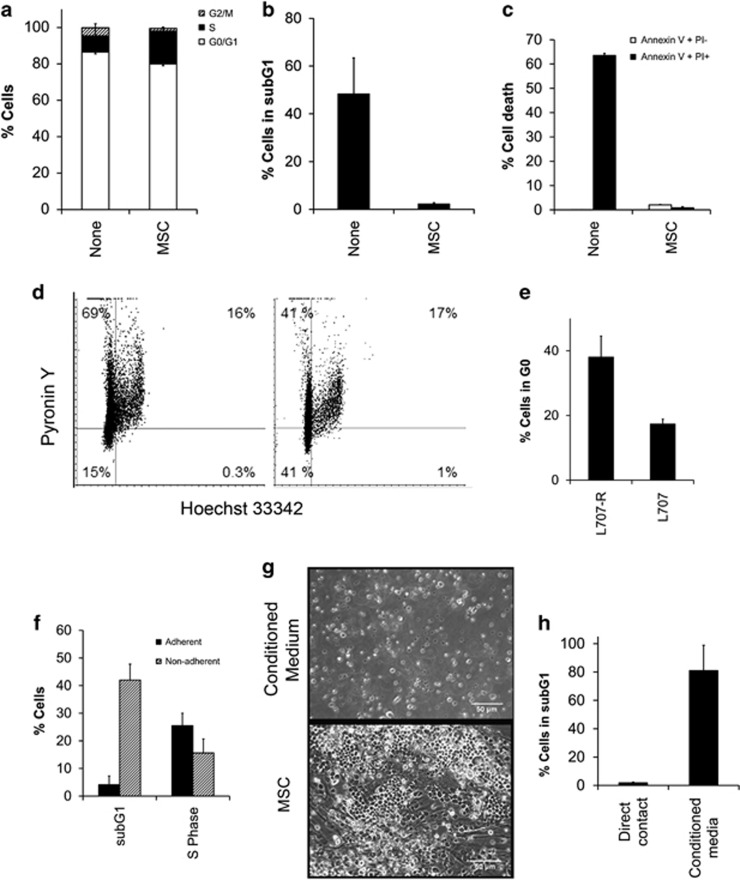
Impact of MSC co-culture on viability and cell cycle distribution of B-ALL cells from patient L707. (**a**) Cell cycle analysis of L707 (E2A/HLF) B-ALL cells. Also see Supplementary Figure S7 for other samples. (**b**) Fluorescence-activated cell sorting (FACS) analysis of subG1 fractions of ALL cells. Also see Supplementary Figure S7 for other samples. (**c**) Apoptosis in L707 cells analysed by Annexin V/propidium iodide staining. (**d**) Detection of G0 ALL cells by Hoechst 33342/Pyronin Y staining. Left panel=None; Right panel=MSC (**e**) Quantitation of L707 relapse and presentation cells in G0 phase. (**f**) Analysis of cell cycle distribution of L707 cells in suspension and adhered to MSC layer. (**g**) Phase-contrast photographs of ALL cells in MSC-conditioned medium and in direct co-culture with MSCs. (**h**) FACS cell-cycle analysis of subG1 ALL cells cultured with MSCs or in MSC-conditioned medium. *N*⩾3 for all experiments shown; error bars, s.d.

Cell cycle analysis showed that co-culture on MSCs caused a twofold increase in ALL cells being in the S phase and a 20-fold reduction in subG1 cell numbers indicating a substantial inhibition of apoptosis (Figures 2a–c and Supplementary Figures S1 and S7). Interestingly, closer inspection of the cell cycle distribution of treatment relapse ALL cells on MSCs using combined Hoechst/Pyronin Y staining revealed a subset of >35% ALL cells being in G0, suggesting that MSCs may support dormant leukaemic cells (Figure 2d). Furthermore, when comparing the matched diagnostic and relapse sample of patient L707, we observed a more than twofold higher fraction of G0 cells in the relapse (Figure 2e).

A more detailed examination showed that this effect is limited to ALL cells adhering to the MSC layer. Nonadherent ALL cells showed subG1 and S-phase distributions similar to ALL cells in standard suspension culture (Figure 2f), that is, a high rate of apoptosis. Notably, MSC-conditioned medium was not sufficient to promote growth of primograft ALL, indicating that direct cell–cell contact is required for the propagation of patient ALL cells (Figures 2g and h and Supplementary Figure S8).

**Figure 3:**
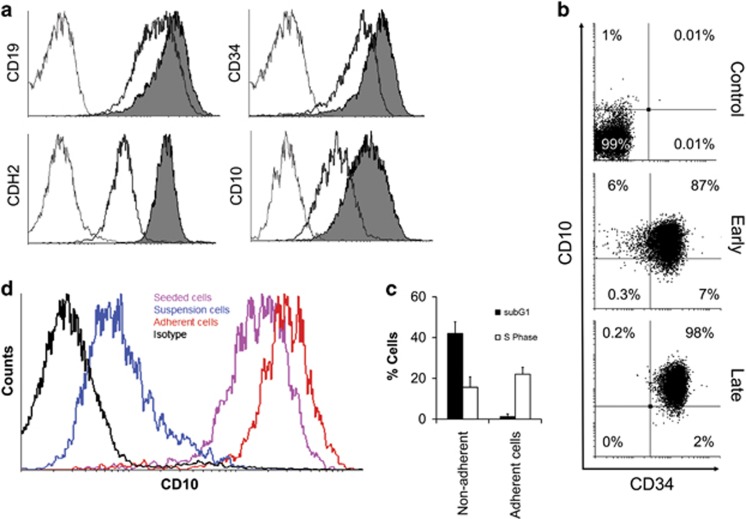
MSC co-culture preserves the ALL immunophenotype. Fluorescence-activated cell sorting (FACS) analysis of CD19, CD34, CD10 and N-cadherin (CDH2) expression. Grey, isotype control; black, nonadherent; grey fill, adherent. (**b**) FACS analysis of CD10 and CD34 expression on L707 ALL cells following 30-day co-culture on MSCs. (**c**) Cell-cycle analysis of suspension ALL cells compared with adherent cells. Here adherent refers to previously suspension ALL cells that adhered when transferred on to a fresh MSC layer. (**d**) FACS analysis of CD10 surface expression on L707 ALLs in suspension monocultures and in co-cultures on MSC. *N*⩾3 for all experiments shown; error bars, s.d.

Most importantly, adherence to MSC affects the expression of genes likely to be involved in cell–cell communication. Surface expression of CD34 (a cell surface glycoprotein involved in cell adhesion), Cadherin 2 (CDH2; a transmembrane gene involved in the formation of adherens junctions) and CD10 (a cell surface enzyme with metalloendopeptidase activity) was between two- and five-fold higher in adherent than nonadherent ALL cells (Figure 3a and Supplementary Figure S2). Some nonadherent ALL cells can adhere to MSCs upon transfer to a fresh monolayer and start to proliferate (Figure 3c).

In addition, long-term ALL expansion over 4 weeks retained the expression of CD10 and CD34 (Figure 3b), unlike in previously published studies where altered immunophenotype in ALL cells has been documented *in vitro*.^35^ Importantly, we observed a drastic reduction in CD10 levels in suspension cultures as opposed to the ALL cells on MSC that continually retained CD10 expression over a 4-week period (Figure 3d). This is consistent with previous reports documenting loss of CD10 in suspension cultures and its expression to correlate with ALL progression *in vivo*.^39, 40^

These combined findings indicate that direct cell–cell contact with MSCs is required to maintain and propagate ALL *in vitro* and that a fraction of the ALL cells in a suspension retain the ability to reinitiate growth when brought back into contact with MSCs.

### MSCs support long-term proliferation of five clinically distinct ALL samples without altering clonal composition

**Figure 4:**
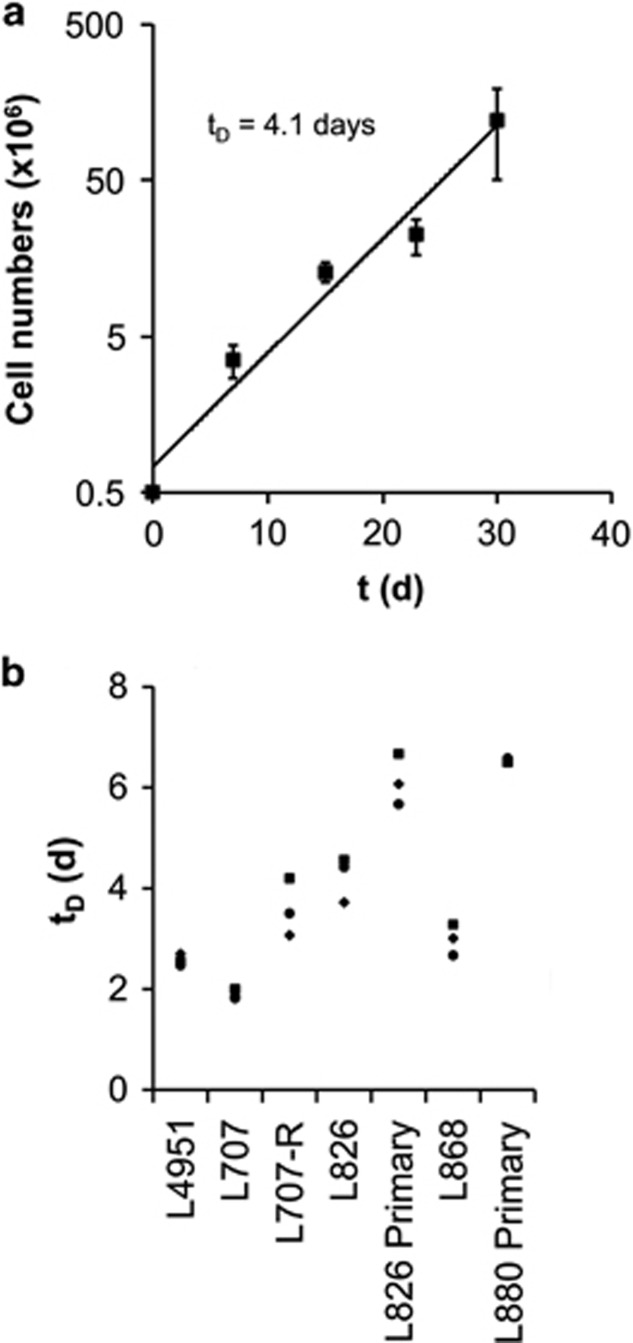
Growth kinetics of B-ALL cells on MSCs. (**a**) Growth curve of primary L826 ALL cells on MSC over a 4-week period. (**b**) Plot representing population doubling times for long-term culture of five different B-ALL samples on MSCs. *t*_D_=doubling time. *N*⩾3 for all experiments shown; error bars, s.d.

To examine long-term expansion, we serially replated ALL cells on new MSC layers. In these experiments, we studied the proliferation of five primografts (L826; L4951; L707, L868; L754) and two primary samples (L826; L880) representing a spectrum of cytogenetically distinct ALLs (Supplementary Table S1). All samples showed sustained proliferation for at least 20 days when weekly replated on MSC layers (Figure 4a and Supplementary Figure S9). Doubling times ranged from 1.5 to 4 days for primograft ALL cells and 6 to 7 days for the primary ALL samples (Figure 4b).

**Figure 5:**
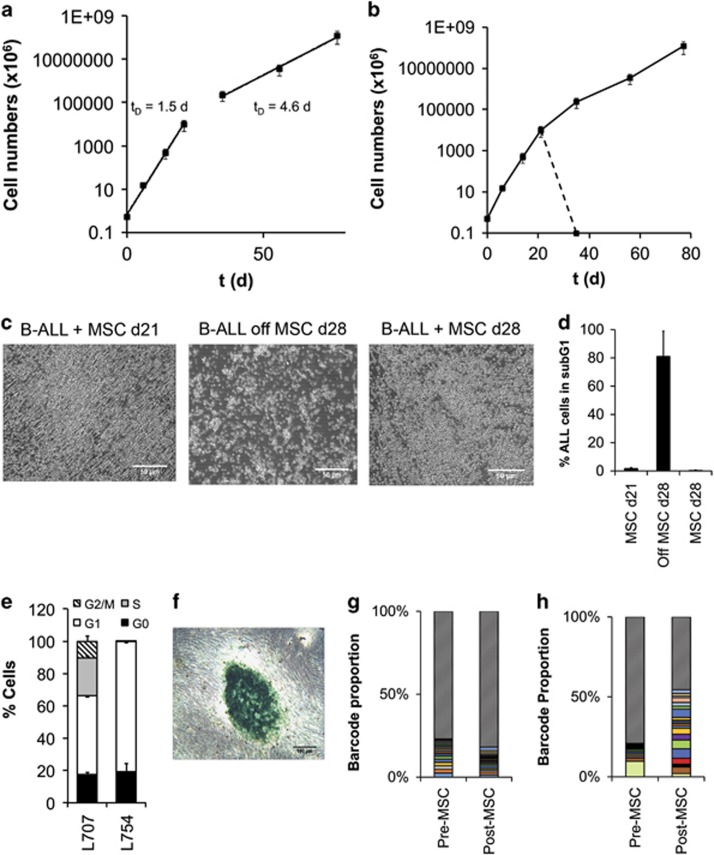
Long-term propagation of ALL cells on MSCs. (**a**) Biphasic growth curve of L707 in long-term co-culture with MSCs. (**b**) Impact of MSC removal on ALL proliferation. L707 cells were either continuously co-cultured on MSCs or after 21 days separated from MSCs (broken line). Cell counts show that B-ALL cells replated on MSCs continued to proliferate, whereas the suspension cells died out within a week. (**c**) Phase-contrast photograph showing B-ALL cells on MSC before feeder removal, B-ALL cells 7 days following feeder removal and post-MSC-separated B-ALL cells when replated onto fresh MSCs. (**d**) Percentage of L707 ALL cells in subG1 on feeders at days 21 and 7 days after removal from the MSC feeder at day 28. (**e**) Cell-cycle analysis of L707 and L754 cells after 5 weeks of co-culture with MSCs. G0 cells were quantified by Hoechst 33342/Pyronin Y G0 staining. (**f**) Senescence-associated β-galactosidase staining of L754 colonies following week 5 of co-culture on MSCs. *N*⩾3 for all experiments shown; error bars, s.d. (**g**, **h**) Clonal composition of barcoded L4951 and L707 cells prior (baseline) and after long-term (4–6 weeks) expansion on MSCs. Each colour represents a distinct clone (L4951=17, L707=23 clones both pre- and post-MSCs) and hatched area represents remaining rarer clones (L4951>1050, L707>550 clones both pre- and post-MSCs).

Selected primograft samples, including L707, could be maintained for more than 80 days in continuous culture, indicating a potentially unrestricted growth potential under these conditions (Figure 5a). ALL cells ceased proliferating when separated from the MSC feeder layer and entered apoptosis as indicated by a substantial increase in subG1 cells and Annexin V+ PI+ cells. In contrast, cells continued proliferation when maintained on MSC feeders (Figures 5b–d and Supplementary Figure S10). These data show that it is possible to maintain primary and primograft ALL cells in tissue culture for prolonged periods of time, without generating cell lines that are able to proliferate independent of feeder support. However, for some leukaemias, prolonged culture on MSCs led to decelerated cell growth (L707) whereas other samples (L754; L880; L826; 19578), although remaining viable, completely ceased proliferation after 20 days and expressed senescence-associated β-galactosidase (Figures 5e and f).

Cellular barcoding is a process by which cells are tagged by a unique DNA sequence (the ‘barcode') that upon stable integration confers a heritable mark so as to trace clonal progenies.^41^ This clonal analysis technique is commonly used for the purposes of evaluating clonal dynamics such as clonal complexity, clonal dominance and founder effects.^19, 42, 43^ We used this cellular barcoding approach as an *in vitro* tool for clonal tracing so as to evaluate whether such prolonged *ex vivo* expansion would cause any MSC-related bottleneck. L4951-BCR/ABL cells were lentivirally transduced with a custom-made barcoded vector library.^36, 43^ Five million cells containing 897 different barcoded clones were seeded onto MSCs. Following 4 weeks of *in vitro* culture on MSCs, 100% of the barcodes in the baseline sample were retrieved, indicating unaltered clonal complexity on the MSC cultures. In addition, there was no selection of dominant clones (Figure 5g). Additional experiments with L707-E2A/HLF and P929-MLL/AF4 further confirmed retention of a high degree of clonal complexity in addition to unaltered clonal composition at varying barcode complexities (Figure 5h and Supplementary Figure S11). Barcodes that were lost completely following MSC co-culture were already rare (<0.1%) in the baseline sample, suggesting this phenomenon to be a stochastic effect. This further suggests that long-term expansion of ALL on MSCs does not significantly limit clonal complexity or lead to clonal dominance, making this model an appropriate tool for investigating clonal dynamics *in vitro*.

### Prolonged expansion on MSCs preserves engraftment potential of ALL cells

**Figure 6:**
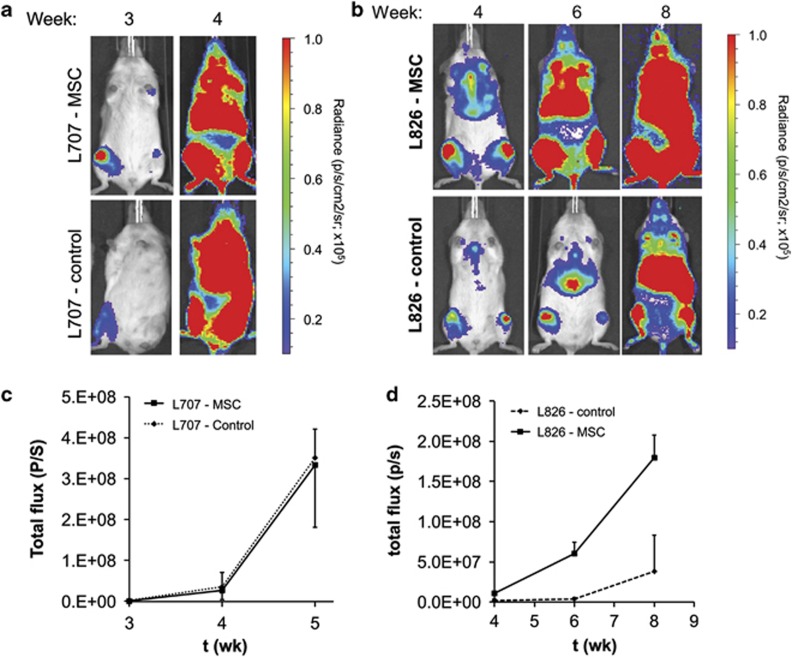
*In vivo* engraftment of patient-derived B-ALL cells following long-term expansion on MSC. (**a**, **b**) *In vivo* imaging of luciferase-tagged L707 (**a**) (*N=*6 experiments) and L826 (**b**) (*N=*4 experiments) B-ALL cells after 8 and 4 weeks of MSC co-culture, respectively. Bottom panels show control engraftment of the corresponding frozen primograft. Note the similarity of L707 engraftment in terms of organ spread especially as central nervous system (CNS, spinal) involvement is classically seen with the L707 control samples. (**c**, **d**) Bioluminescence reading of total flux shows comparable patterns in the rate of engraftment between control and B-ALL–MSCs. Error bars, s.d.

Prolonged expansion of primary leukaemic cells frequently impairs their self-renewal capacity and, consequently, their ability to induce leukaemia in immunodeficient mice.^34^ Therefore, we examined whether extended MSC co-culture affects the engraftment potential of primograft cells. We used two primografts (L707 and L826) that had been transduced to stably express luciferase.^6, 36^ Consequently, engraftment of these primografts can be monitored *in vivo* using bioluminescent imaging. For the t(17;19)-positive L707 sample, co-culture on MSC feeder for 8 weeks did not diminish the engraftment potential when compared with uncultured primograft cells (Figures 6a and c). In both settings, mice showed the characteristic leukaemic spread including infiltration of spleen, spine, sternum and calvaria within 4 weeks after transplantation (Figures 6a and c and Supplementary Figure S12). The t(4;11)-positive ALL primograft L826 was cultured on MSC for 4 weeks prior transplantation. These cells showed an even accelerated leukaemic engraftment and spread compared with directly thawed from frozen starting material, possibly because of a higher viability of the ALL cells following MSC co-culture as compared with the freshly thawed sample (Figures 6b and d). This retention of leukaemia-initiating potential demonstrates that prolonged culture on MSCs maintains the self-renewal capacity of patient-derived ALL samples.

### MSC co-culture allow bioluminescence-based drug combination screens with patient-derived ALL cells

**Figure 7:**
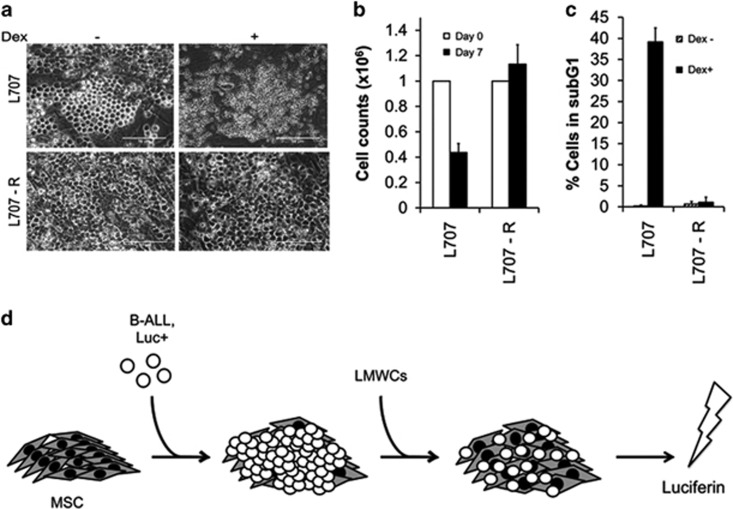
Patient-derived ALL drug screen. (**a–c**) Phase-contrast photographs (**a**), cell counts (**b**) and cell-cycle analysis (**c**) of L707 presentation and relapse (L707 and L707R) cells with and without Dexamethasone. *N=*3 experiments; error bars, s.d. (**d**) A scheme of testing low-molecular-weight compounds (LMWCs) in a bioluminescent ALL–MSC co-culture system. Primograft cells were lentivirally transduced to stably express firefly luciferase.

Finally we examined the suitability of the ALL/MSC− co-culture system for the evaluation of therapeutic compounds. In an initial experiment, we asked whether this system faithfully reflects clinically observed drug responses. We treated a matched presentation/relapse pair of the fatal E2A/HLF-positive translocation samples (L707 and L707R, respectively) with dexamethasone (Figure 7a). At diagnosis, the corresponding patient from whom these samples were derived from responded to dexamethasone, but gained resistance upon relapse through loss of the steroid receptor. Both samples proliferated on MSC layers (Figure 1d). Treatment with dexamethasone for 1 week reduced cell numbers of the presentation sample more than twofold, whereas the relapse sample continued to proliferate (Figure 7b). This was paralleled by an 80-fold increase in apoptosis in the presentation sample, whereas the relapse showed only a negligible induction of apoptosis (Figure 7c). Therefore, for this patient and for treatment with dexamethasone, the *in vitro* co-culture system recapitulates clinically observed treatment responses.

**Figure 8:**
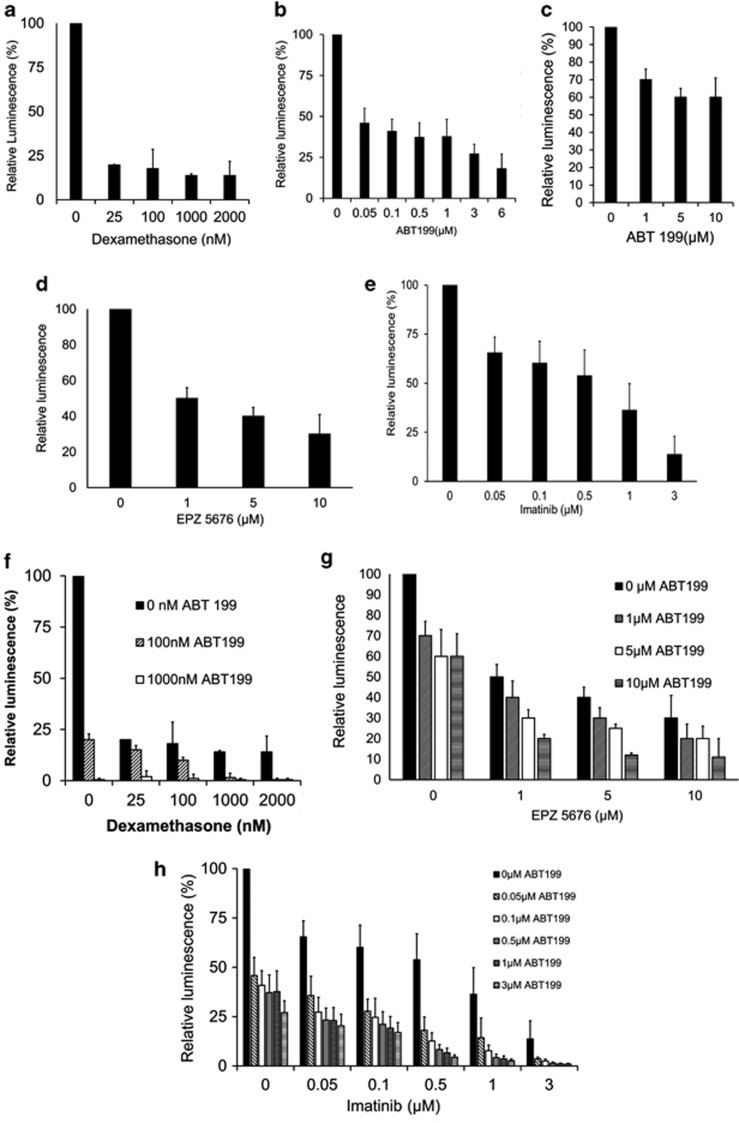
Bioluminescent drug combination screen using patient-derived ALL. (**a–e**) Dose-related response as depicted by decreasing bioluminescence using single-drug targeted pharmacotherapy. The 100% relative luminescence refers to untreated control. (**a**) Dexamethasone on L707-E2A/HLF, (**b**) BCL2 inhibitor ABT-199 on L4951-BCR/ABL and (**c**) L826-MLL/AF4, (**d**) DOT1L inhibitor EPZ5676 on L826-MLL/AF4 and (**e**) imatinib on L4951-BCR/ABL. (**f–h**) Synergistic drug combinations. (**f**) Dexamethasone/ABT-199 (combination index (CI)=0.32 at half-maximal effective dose (ED_50_)) on L707-E2A/HLF, (**g**) EPZ5676/ABT-199 (CI=0.39 at ED_50_) on L826-MLL/AF4 and (**h**) imatinib/ABT-199 (CI=0.88) on L4951-BCR/ABL. *N=*3 for all experiments shown; error bars, s.d.

To improve the co-culture assay and create an up-scalable tool for high-throughput screening, we started to use bioluminescence-labelled primary ALL cells^36^ that allows live monitoring of cell numbers in 96-well plates in response to single and combinations of candidate anti-leukaemic drugs (Figure 7d). In the first instance, we assessed proven drug combination response on our platform to corroborate its credibility in predicting documented drug efficacies. MSC feeders were established in 96-well plates and seeded with dexamethasone-sensitive L707-E2A/HLF cells stably expressing luciferase. The co-cultures were then exposed to increasing concentrations of dexamethasone alone (Figure 8a) and in combination with the BCL2 inhibitor ABT-199 (Figure 8f). Cell numbers were monitored through bioluminescence. Even at the lowest concentration, dexamethasone reduced cell counts by fivefold. Furthermore, consistent with existing literature,^44^ we confirm synergism of dexamethasone/ABT-199 (combination index (CI)=0.32) in our MSC bioluminescence-based drug assay. We repeated the drug-combination experiment with another primary sample (L826-MLL/AF4) and in accordance with reported literature^45^ confirmed a combination of DOT1L inhibitor EPZ5676 and ABT-199 (CI=0.39) to be synergistic in MLL rearranged leukaemia (Figures 8c, d and g).

We and others have previously shown BCL2 to be active in t(9;22) ALL.^6, 46^ In this context we identify drug combination imatinib/ABT-199 to show synergism in BCR/ABL leukaemia (CI=0.88) (Figures 8b, e and h). These data confirm credibility of the bioluminescence MSC-based model as a new experimental platform for medium–high throughput *in vitro* testing of drug combinations and further suggest that the combination of ABT-199 with Imatinib may warrant further investigation for the treatment of very high risk ALL/t(9;22).

## Discussion

In this paper we show that primary and patient-derived ALL cells can be successfully expanded *ex vivo* often over prolonged periods of time, without loss of leukaemic self-renewal potential and while maintaining the multitude of founder clones in these cultures.

MSCs play an important role in the normal haematopoietic and leukaemic bone marrow niche.^47, 48, 49^ In particular, *in vivo* studies have shown that following cytotoxic treatment MSC cooperate with B-ALL cells to create a supportive microenvironment that confers chemoprotection.^48^ Consistent with these *in vivo* and previous *in vitro* studies,^11, 12, 32, 50^ we confirm that freshly sourced low-passage Nestin-positive MSCs are able to provide superior support for long-term *in vitro* expansion of primary and patient-derived lymphoid blasts compared with murine M210B4 or human *TERT*-immortalised MSCs. Through fate mapping we show that hundreds of founder clones establish the leukaemia in these cultures, continue to contribute to the *in vitro* expanding leukaemia over time and maintain the ability to re-establish the disease in immunodeficient mice.

Direct MSC contact is required to support proliferation and viability of B-ALL and cannot be substituted by MSC conditioned medium. A possible mechanism behind this contact-mediated survival may be attributed to the formation of tunnelling nanotubules between the MSCs and B-ALL cells.^51^ Adherence to MSCs is an interactive process leading to the upregulation of key molecules on the surface of the leukaemic blasts that are presumed to play a role in cell–cell contacts. This was shown for the cell adhesion molecule CD34 and, more importantly, for cadherin 2 that is involved in establishing tight adhesion junctions between cells. Another molecule upregulated upon adhesion to MSCs but lost in suspension culture was CD10. CD10 is a cell surface molecule with metalloprotease activity that is able to inactivate a range of peptide hormones and as such expected to regulate signals from the microenvironment. Similar to our observation, CD10 has been shown to be induced and to correlate with progression of ALL *in vivo* while being lost *in vitro* during suspension culture;^39, 40^ it has also been shown to correlate with dissemination and progression of melanoma and other types of cancer.^52^

Interestingly, our cell cycle analyses show that a high number of ALL blasts go into cell cycle arrest (G0) upon direct contact with MSCs. This could be seen as a limitation of this assay as not all ALL samples will continue to proliferate and expand long term; however, it provides a tool to study the mechanisms that regulate quiescence/dormancy of leukemic cells. In summary, the ALL/MSC co-culture system provides an experimentally accessible model to study and dissect key components of the leukaemic niche, including how the niche facilitates chemoprotection.

Drug response validation experiments show that *in vitro* response in the ALL/MSC co-culture assay correlates with clinical and *in vivo* responses, as shown here for steroid and targeted drug-combination sensitivities. Testing novel agents on primary and primografted cells on MSC co-cultures will increase the predictive value for clinical responses as compared with historic suspension culture assays. To further improve the assay system compared with previously published MSC co-culture models^10, 11, 12, 32, 50^ and to create an up-scalable tool for high-throughput screening, we introduced bioluminescence-labelled ALL cells allowing live monitoring of cell numbers in 96-well plates in response to combinations of candidate anti-leukaemic drugs.

Although not all ALL samples grow long term on MSCs, using bioluminescent-labelled patient-derived ALL cells after expansion through immunodeficient mice (primografts), this assay will significantly simplify preclinical drug testing by using a clinically relevant cell source and by improved modelling of the *in vivo* microenvironment while significantly reducing expensive *in vivo* experiments that are currently regarded as gold standard assays for preclinical drug development. Most importantly, as we show that the MSCs preserve the initial multitude of leukaemic founder clones, this assay will allow complex functional RNAi/CRISPR (RNA interference/clustered regularly interspaced short palindromic repeats) screens to study synthetic lethality, thereby facilitating design of novel and clinically effective combination therapies for ALL.
