# Supplementary material for: Long-term in vitro maintenance of clonal abundance and leukaemia-initiating potential in acute lymphoblastic leukaemia
Source: Leukemia. 2016 Jul 10;30(8):1691–700. doi: 10.1038/leu.2016.79 (PMC4980562; doi:10.1038/leu.2016.79)
Supplement: Supplementary Information [file leu201679x1.doc]

**Supplemental Figures:**

**a**


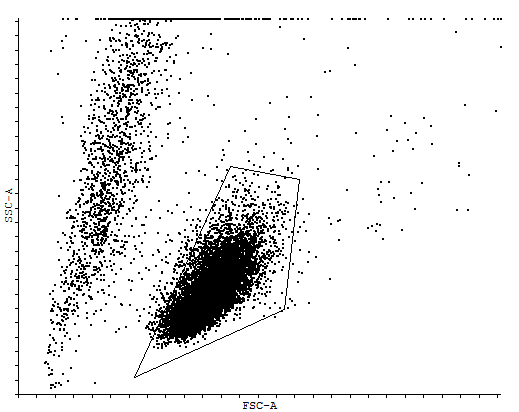


SSC-H

FSC-H


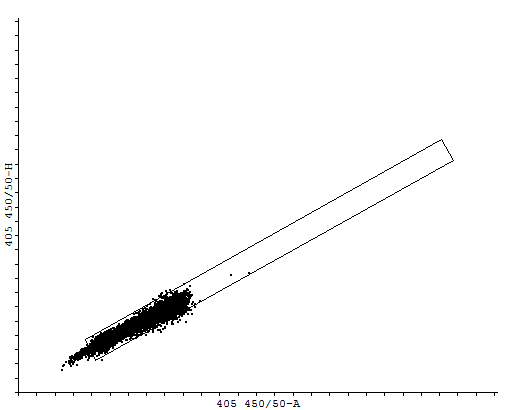


DNA-peak

DNA-area

**B**

Figure S1: Gating to exclude dead cells and doublets for cell cycle analysis

SSC-H

FSC-H

B-ALL cells


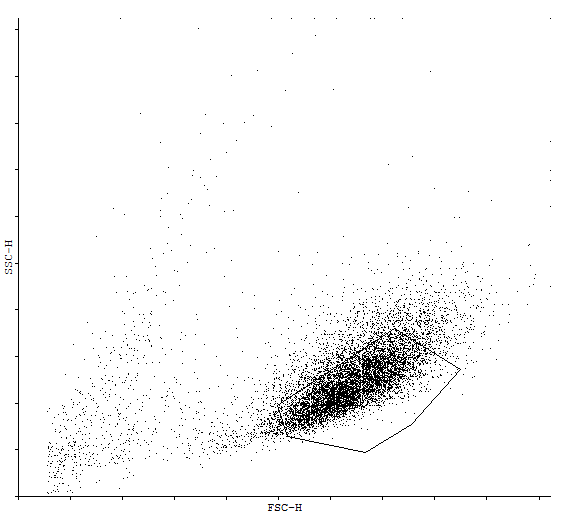


SSC-H

FSC-H

MSC at B-ALL setting


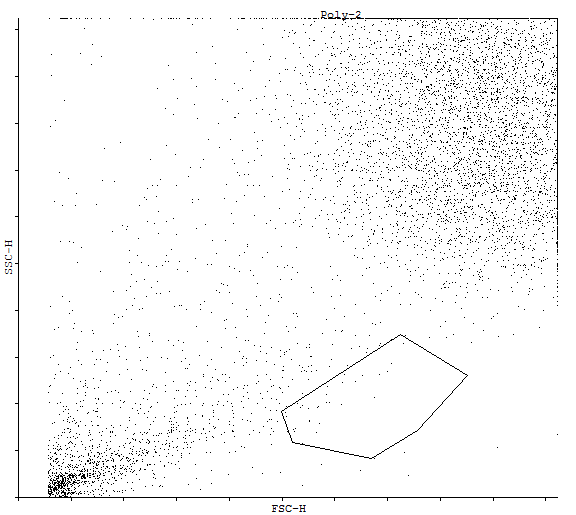


Figure S2: Gating strategy for exclusion of MSC in B-ALL analyses.

**a**


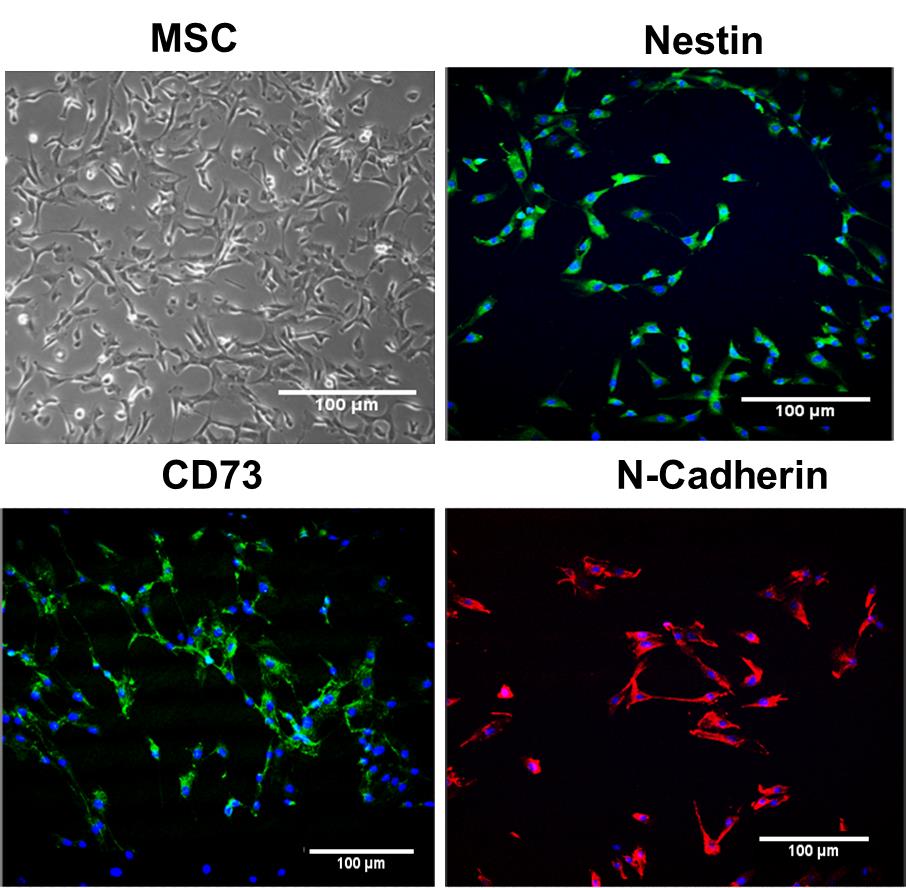


**b**

**c**

**d**


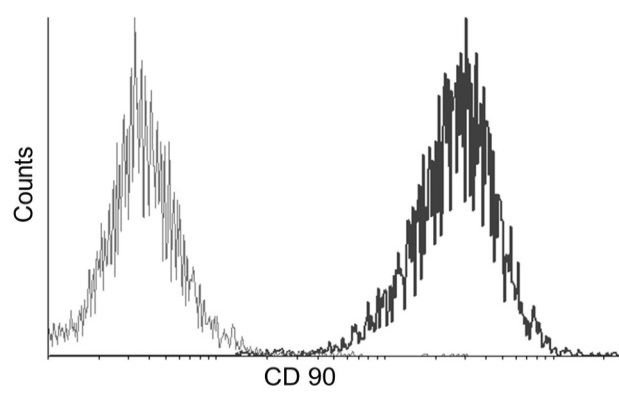

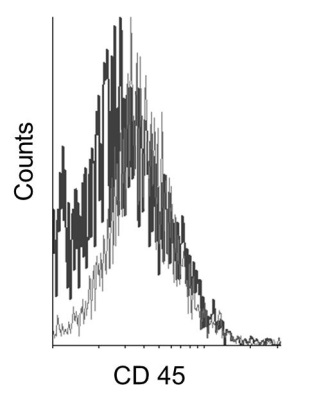

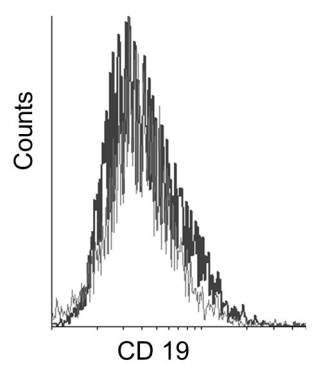


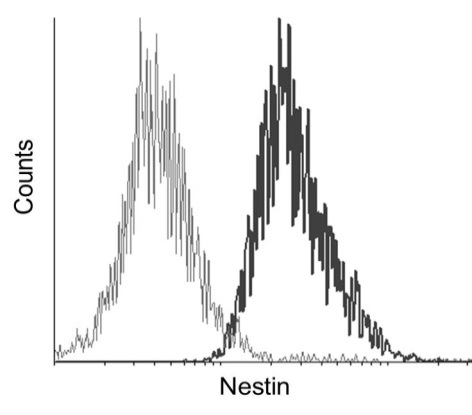

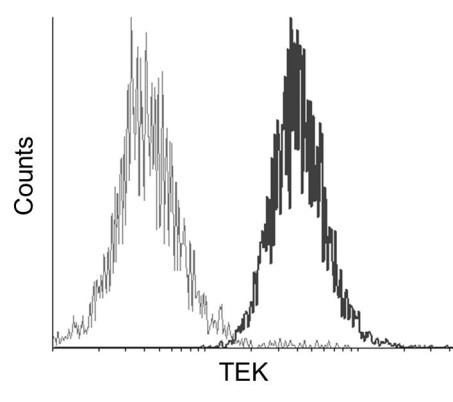


**e**

**f**

Figure S3: Characterisation of MSC. A: Detection of Nestin, CD73 and N-Cadherin expression on MSCs by immunofluorescence. B-F. FACS analysis of MSC. MSC are negative for CD19 (B) and CD45 (C) and are positive for CD90 (D), Nestin (E) and TEK (F). Grey = isotype control. Black = stained MSC. N = 3 experiments

**a**


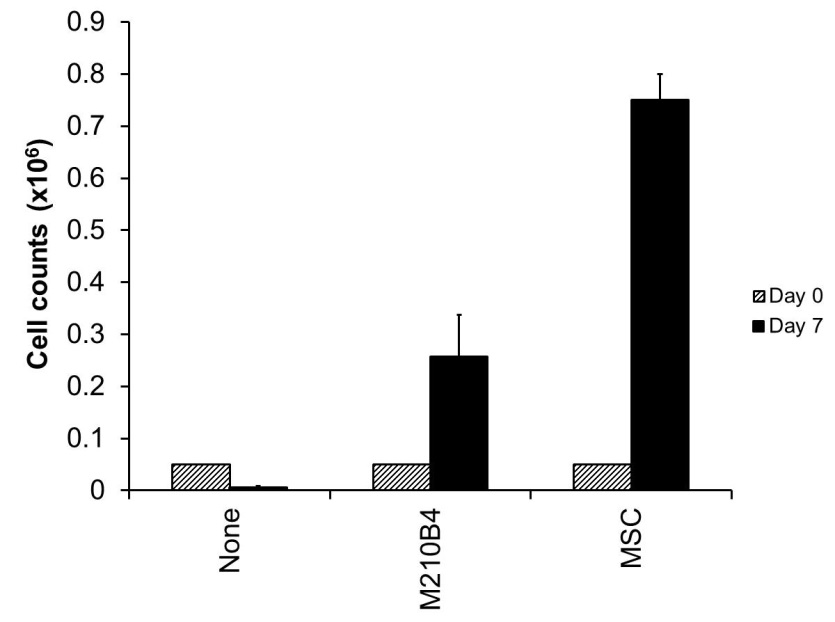


**b**


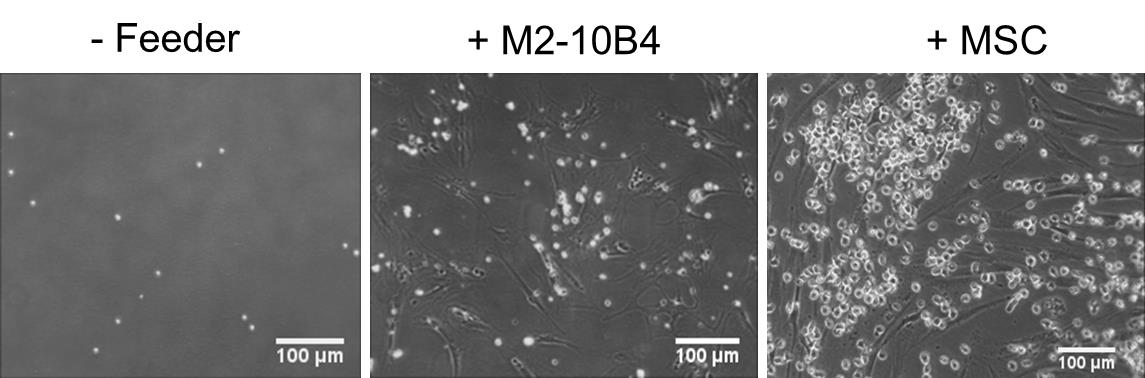


Figure S4: MSC support REH ALL cells in very low cell density. A: MSC support nearly four population doublings of REH at 5,000 cells / ml in 4% FBS whilst M210B4 support only 2 population doublings of REH in the same culture conditions. N = 3 experiments; error bars, SD. B: Phase contrast photographs of REH cells without feeder, murine M2-10B4 and human MSC feeder layers.


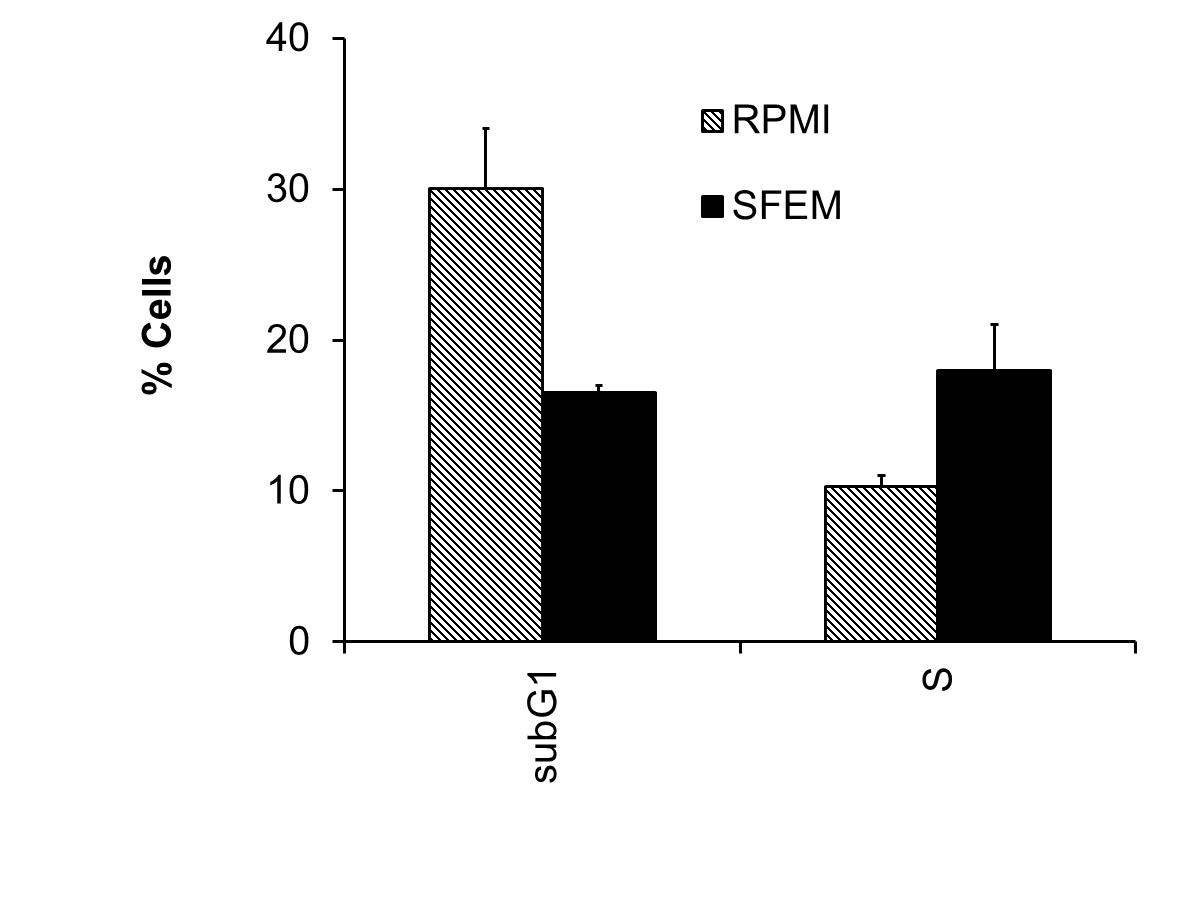


Figure S5: FACS analysis demonstrating that B-ALL co-cultured with MSC in SFEM show a 50% reduction in subG1 phase and a two-fold increase in S phase. N = 3 experiments; error bars, SD.

**A**

**b**


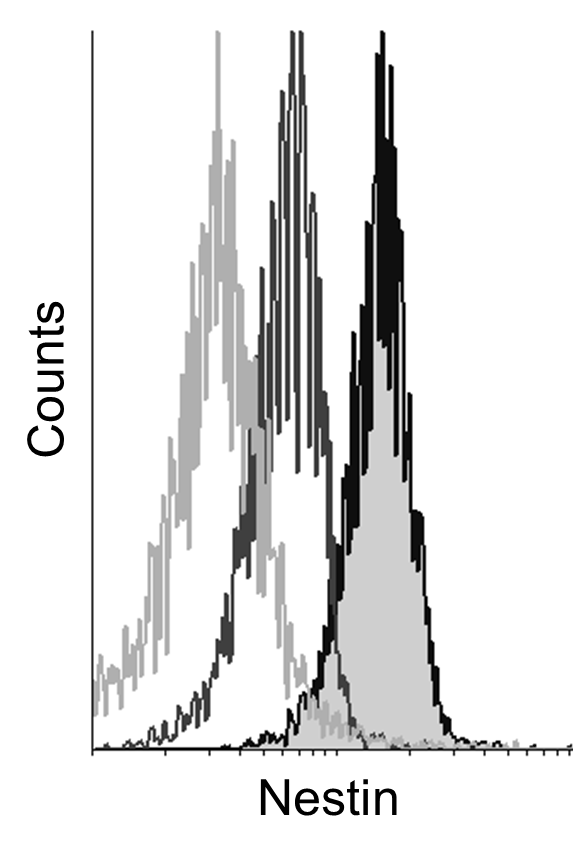


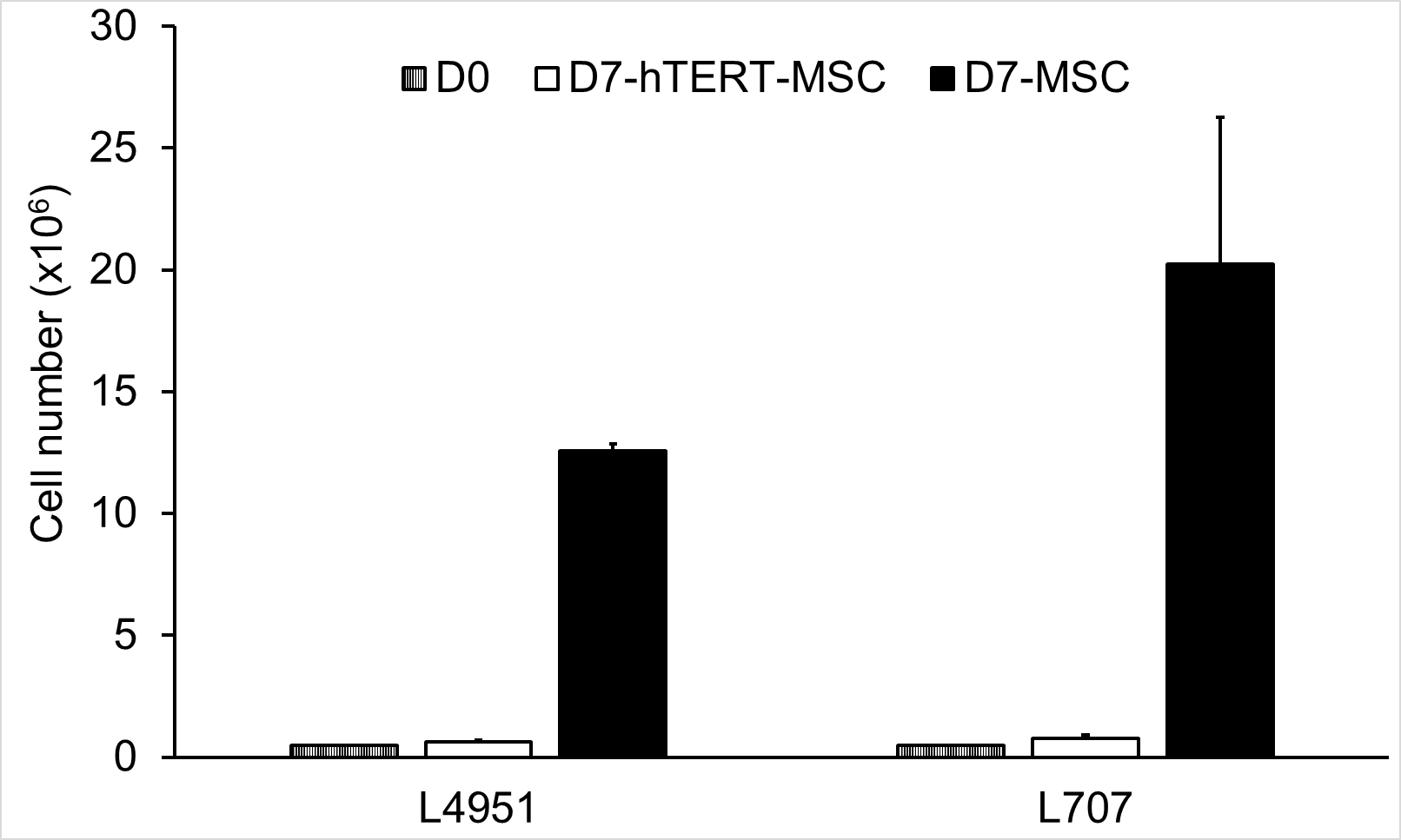


Figure S6. A. hTERT and primary MSC both maintain 100% viability of primary ALL. Expansion of ALL on primary MSC is greater than on hTERT-MSC B. Primary MSC express higher levels of Nestin than hTERT-MSC. Grey = isotype control. Black = hTERT-MSC. Black with grey-fill = primary MSC. N = 3 experiments


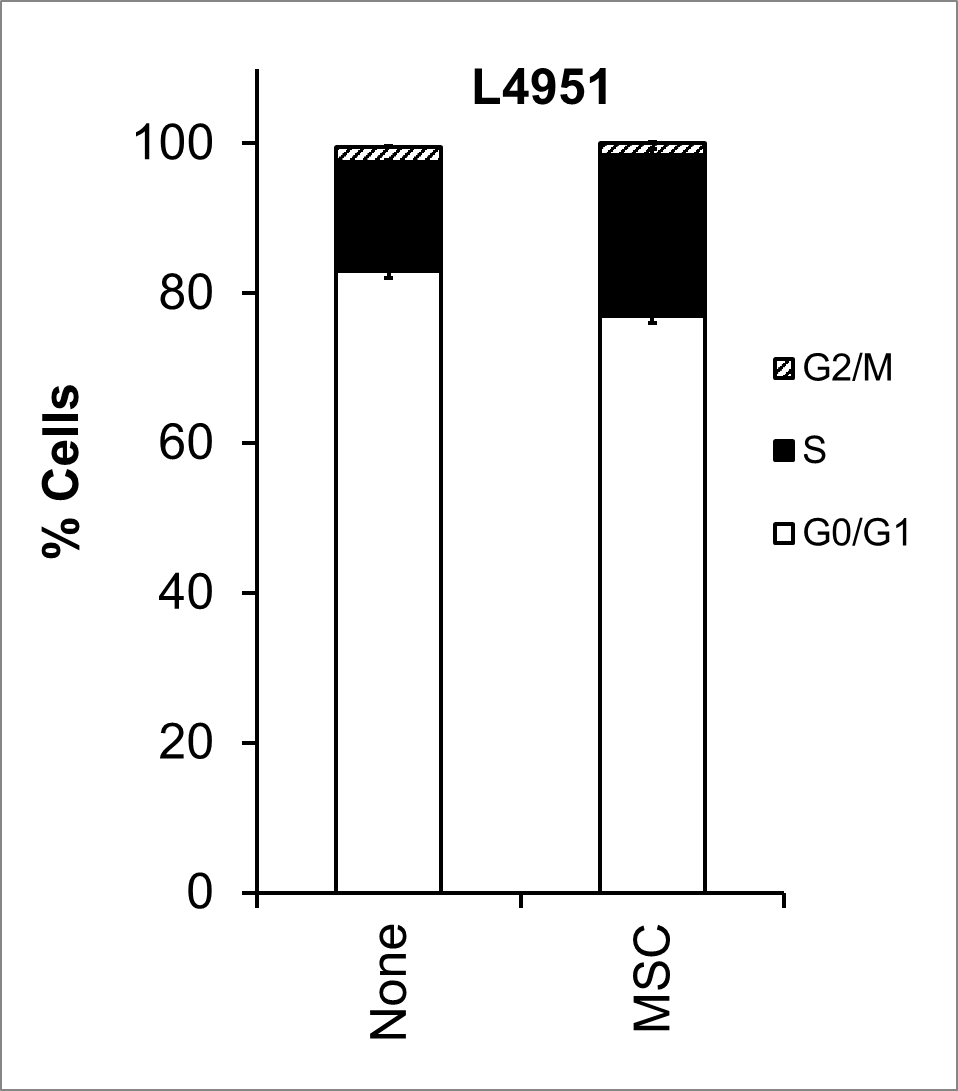

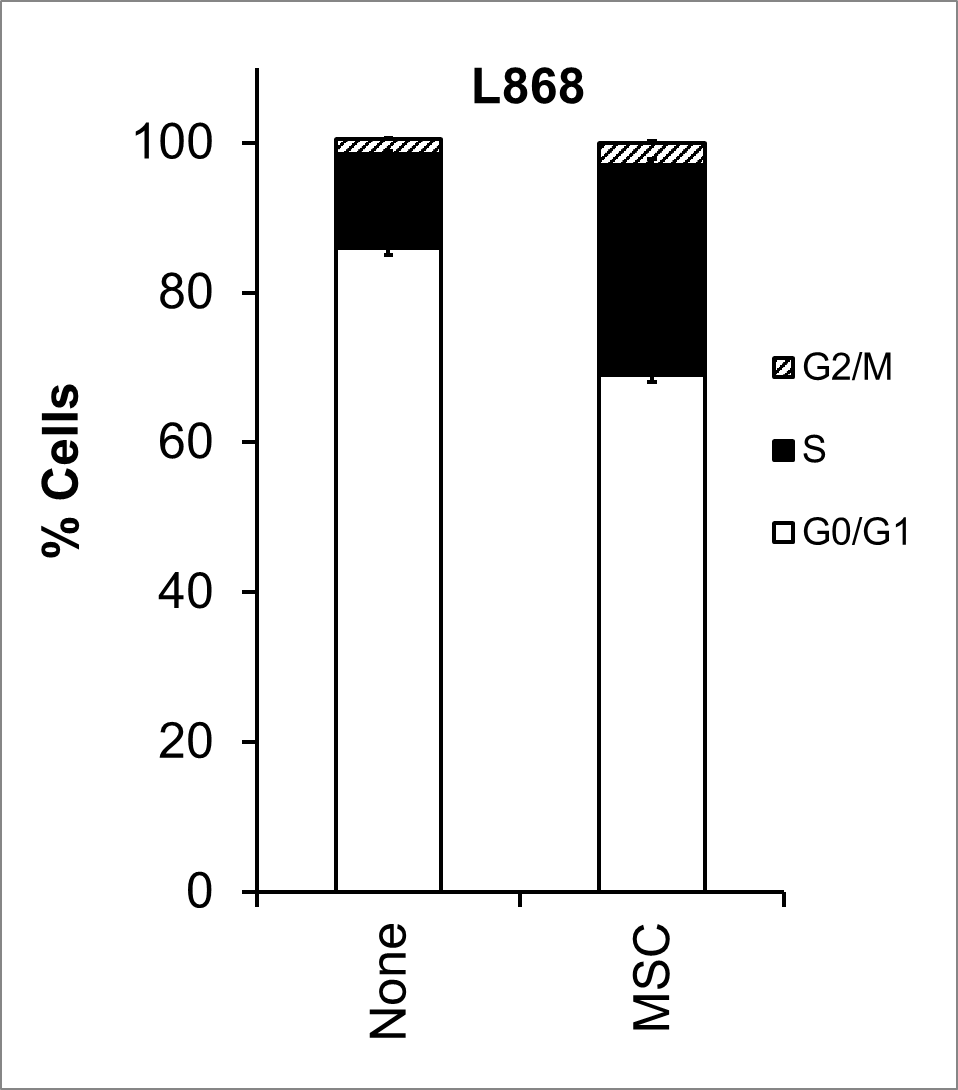

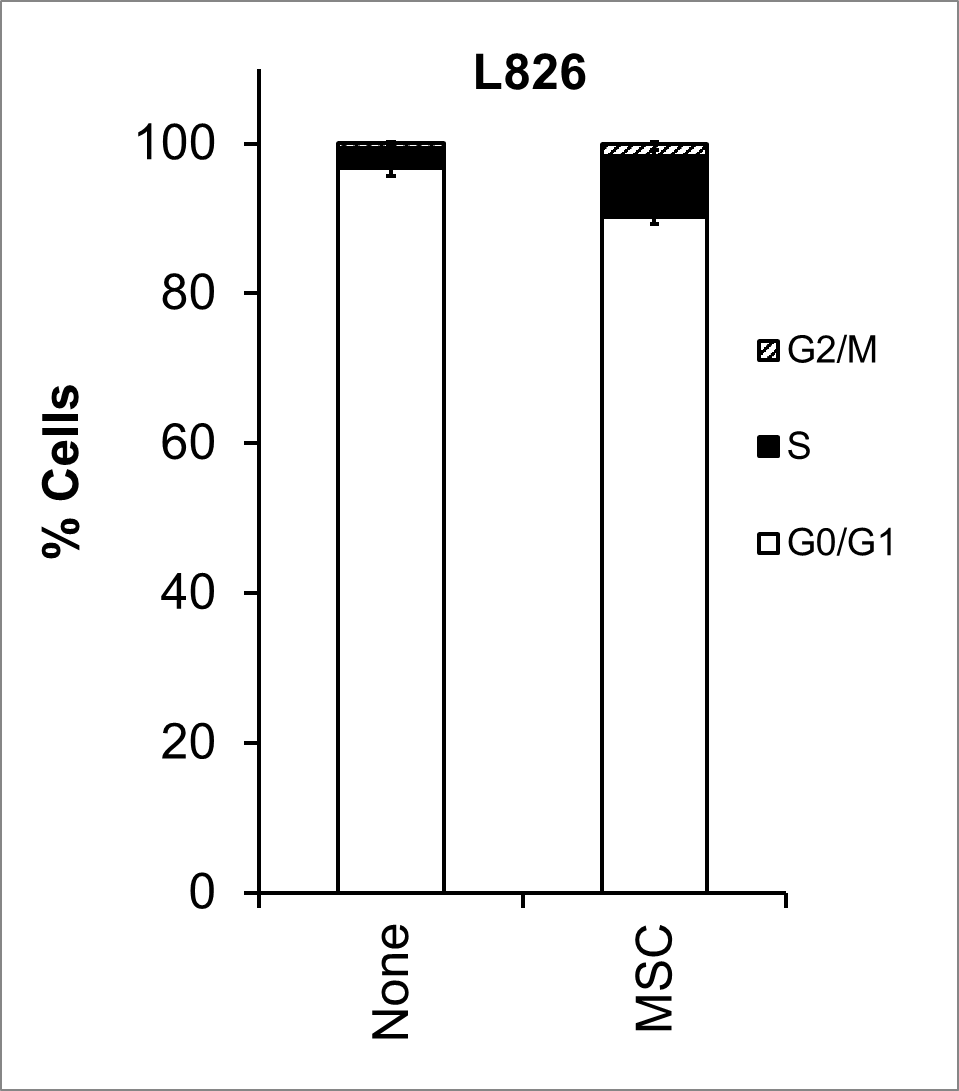


**A**

**B**

**C**

**D**

**E**

**F**


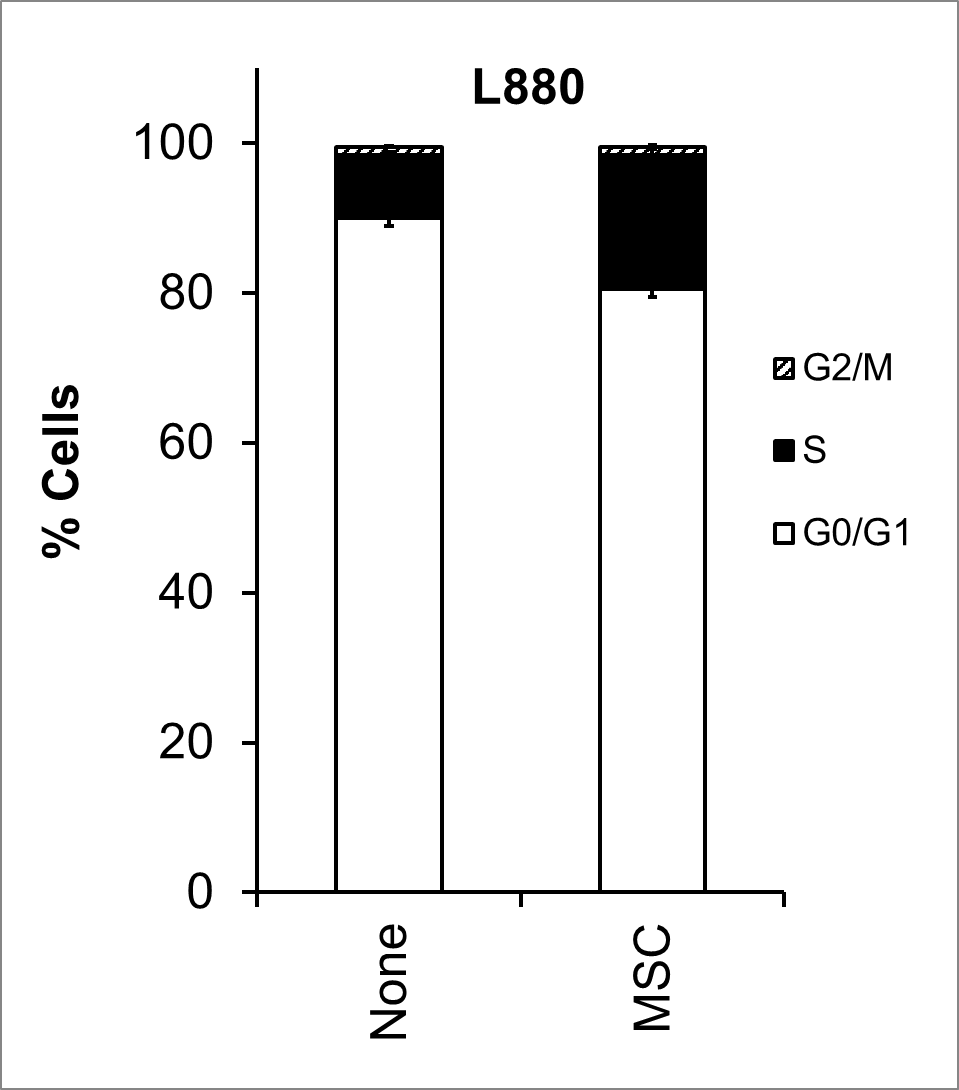


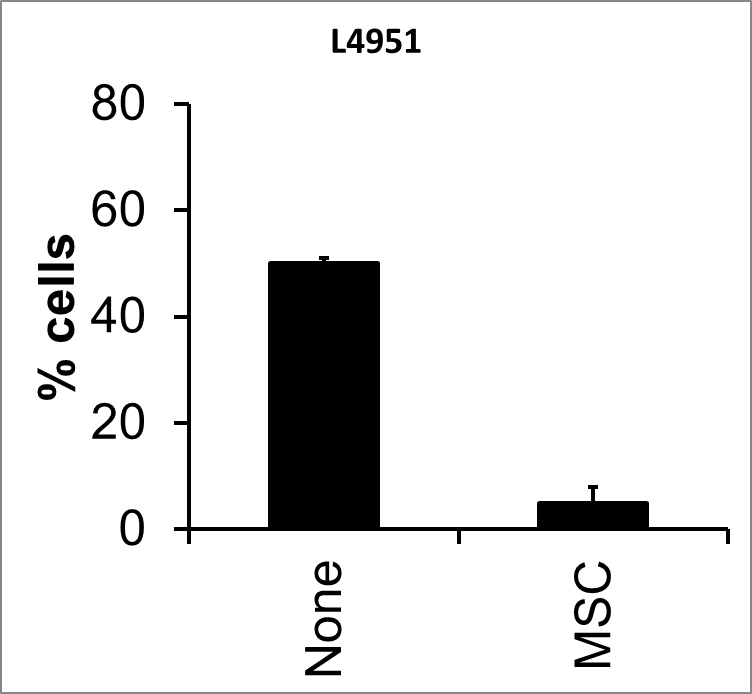

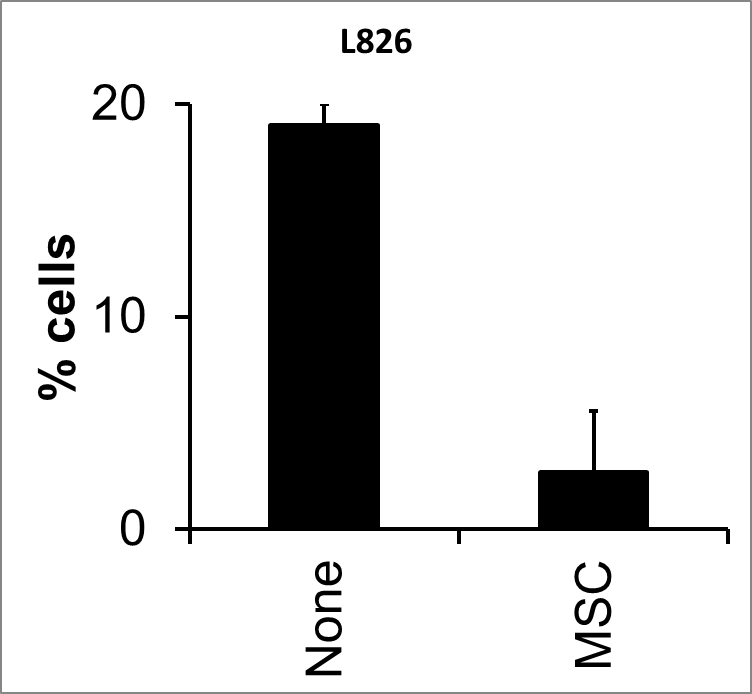


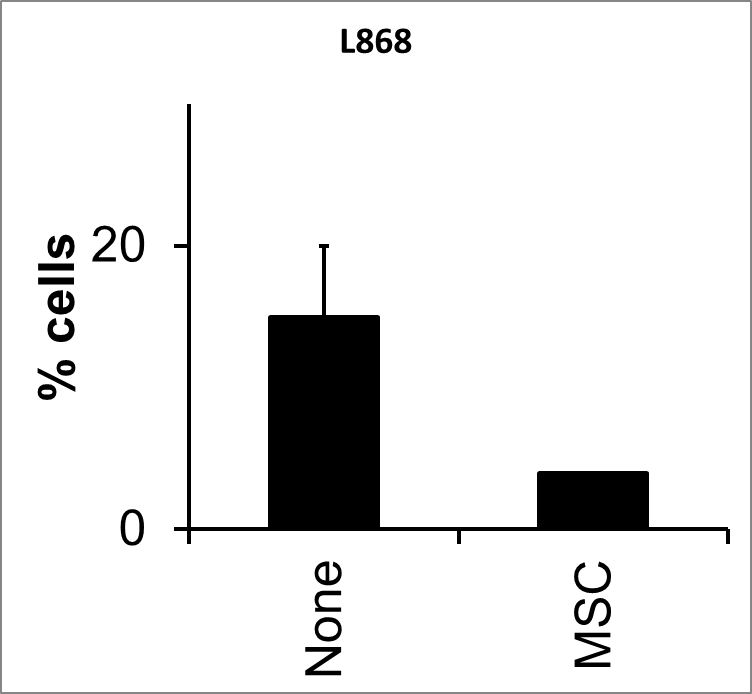

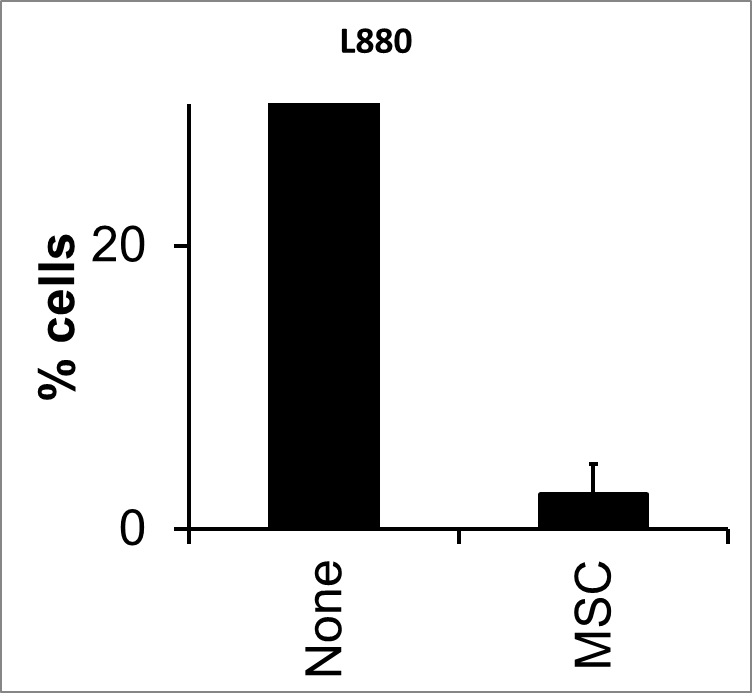


**G**

**H**

**H**

Figure S7: MSC maintain viability and support proliferation of patient-derived B-ALL. A-D. Cell cycle distribution of B-ALL cells from patients L826 (MLL/AF4), L4951 (BCR/ABL), L868 (iAMP21) and L880 (MLL/AF9) with and without MSCs. Nearly twice as many B-ALL cells are in S phase when co-cultured with MSCs. N = 3 experiments; error bars, SD. E-H. FACS cell cycle analysis of subG1 (i.e. apoptotic) ALL cells with and without MSCs. N = 3 experiments; error bars, SD.

**a**


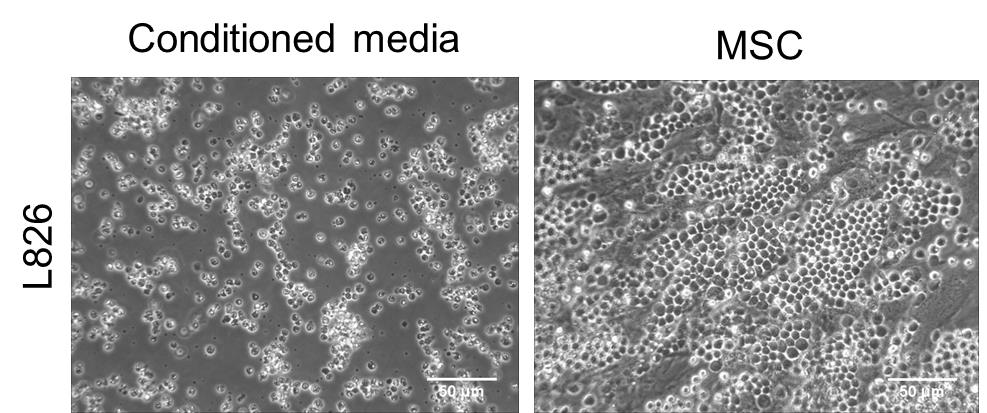


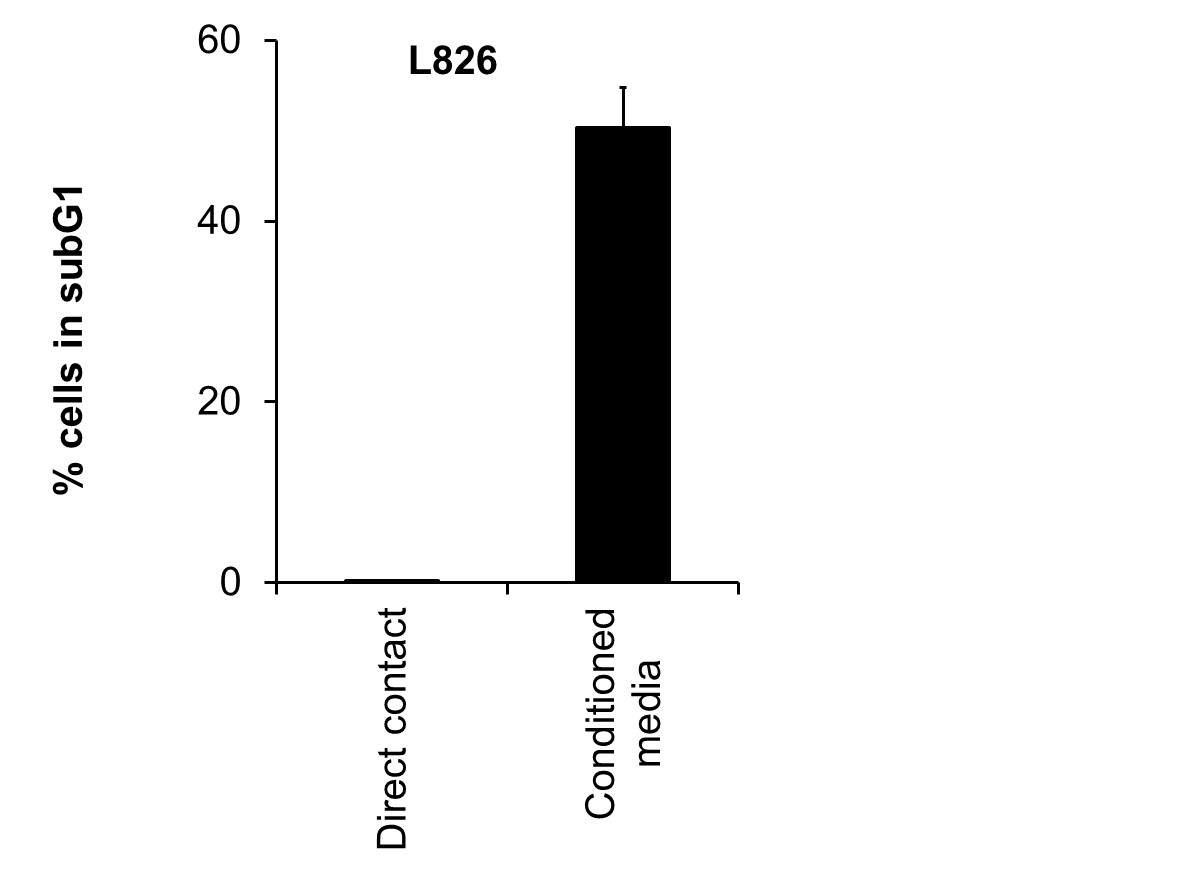

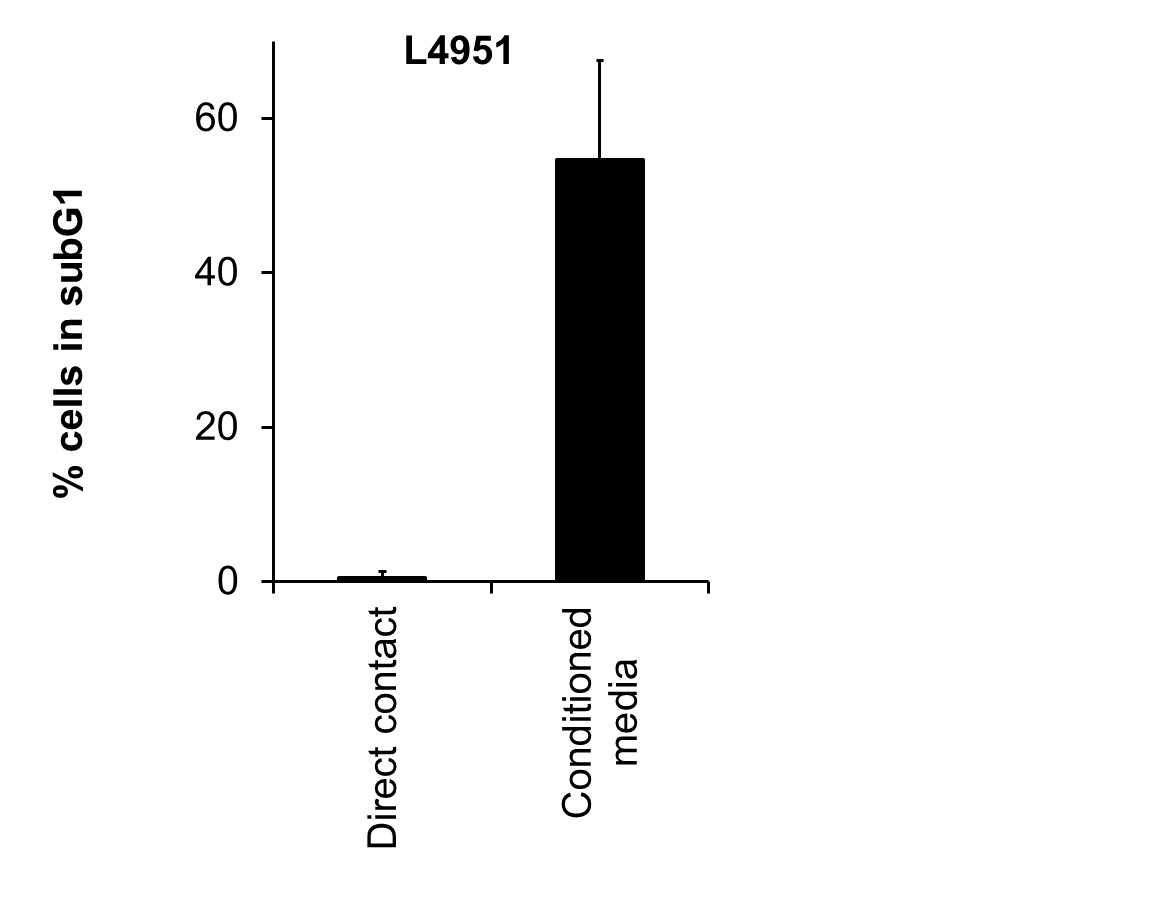


**B**

**C**

Figure S8: B-ALL do not survive in conditioned media. A: Phase Contrast photographs of ALL cells in MSC-conditioned medium and in direct co-culture with MSCs. B, C: FACS cell cycle analysis of subG1 (i.e. apoptotic) L826 (B) and L4951 (C) ALL cells cultured with MSCs or in MSC-conditioned medium. N = 3 experiments; error bars, SD.


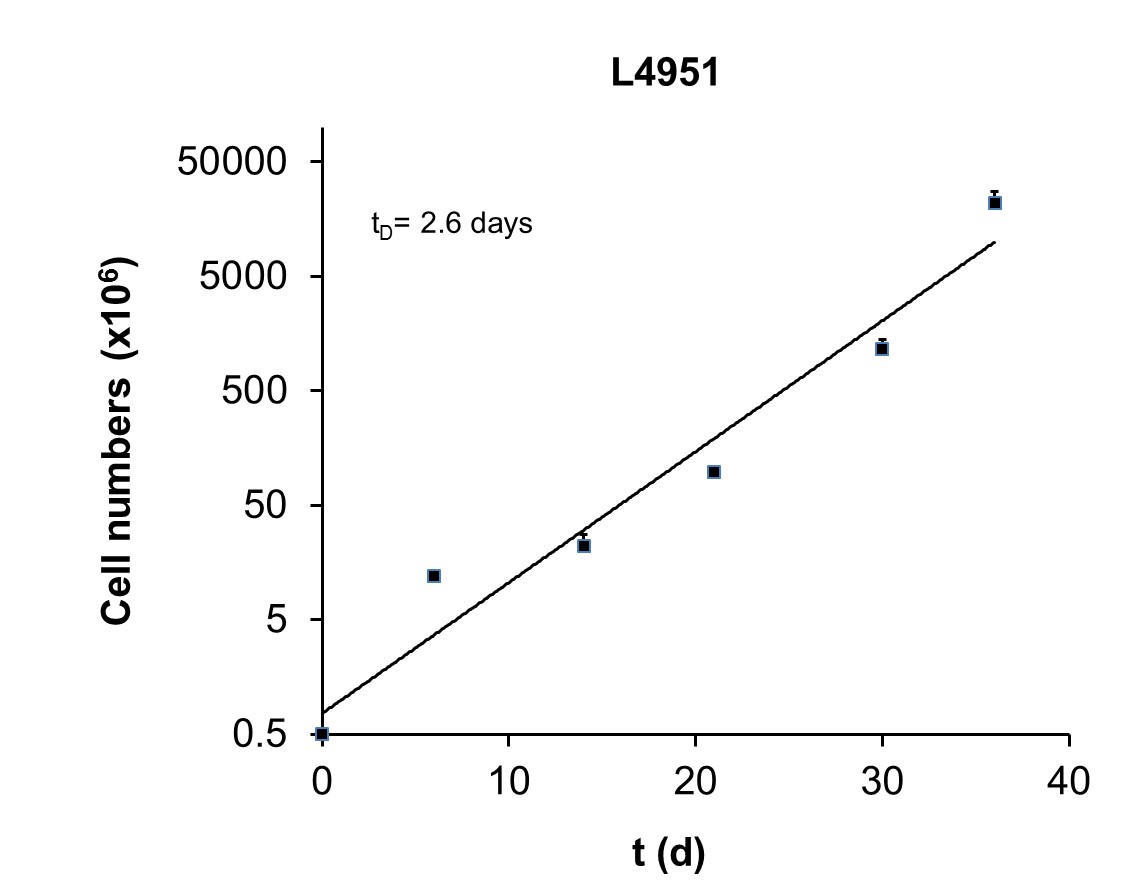


**a**

**B**


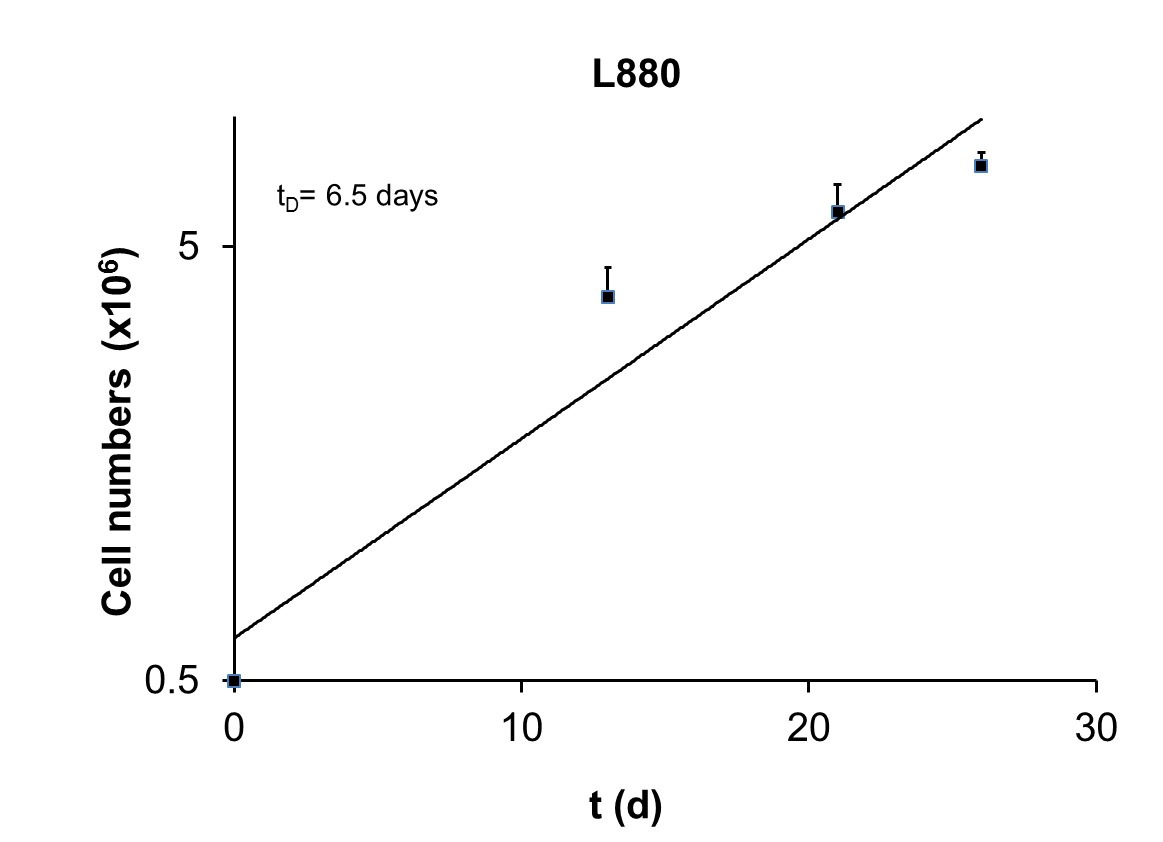


**C**


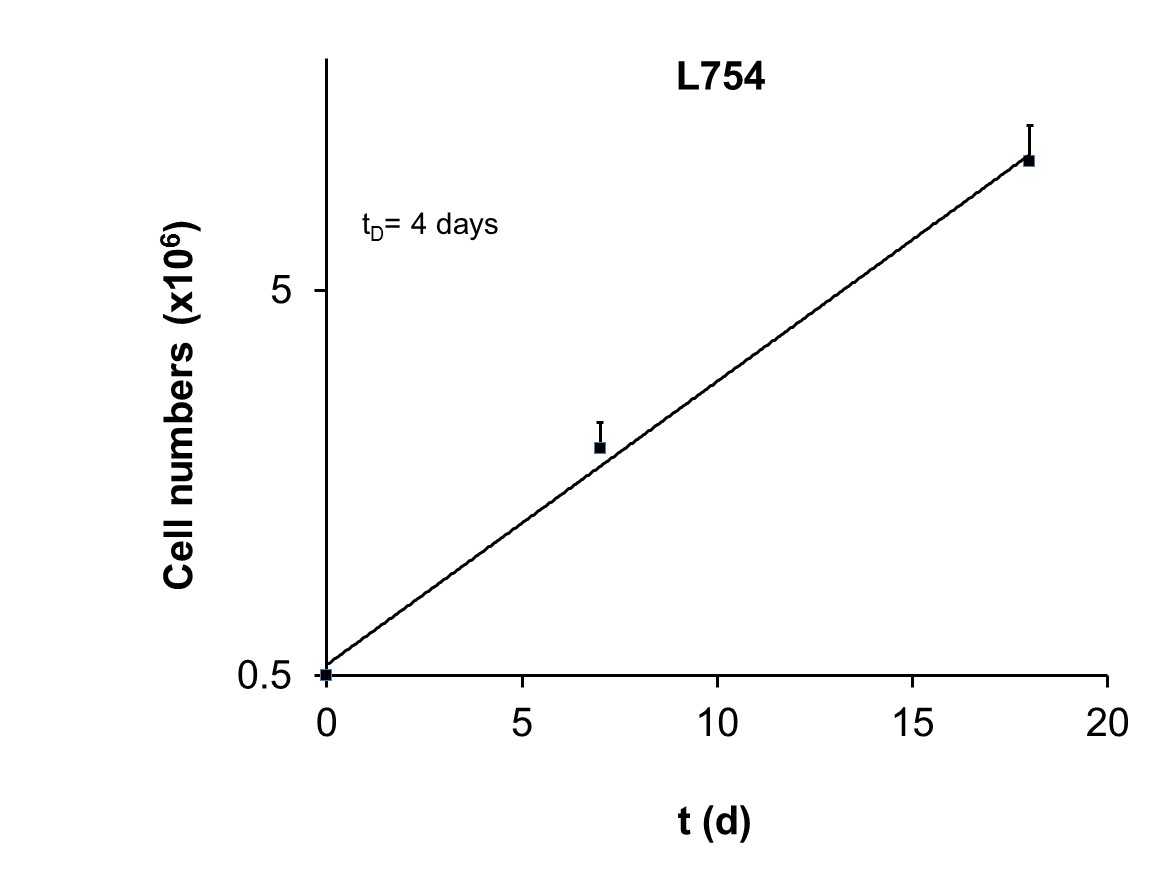


Figure S9: B-ALL cell numbers on MSC during long-term *in vitro* culture. The graphs show growth curves for BCR/ABL translocation/Philadelphia chromosome positive primograft (A), primary sample from a MLL/AF4 ALL patient (B) and a high hyperdiploid primograft sample (C). t_D_, doubling time. N = 3 experiments; error bars, SD.


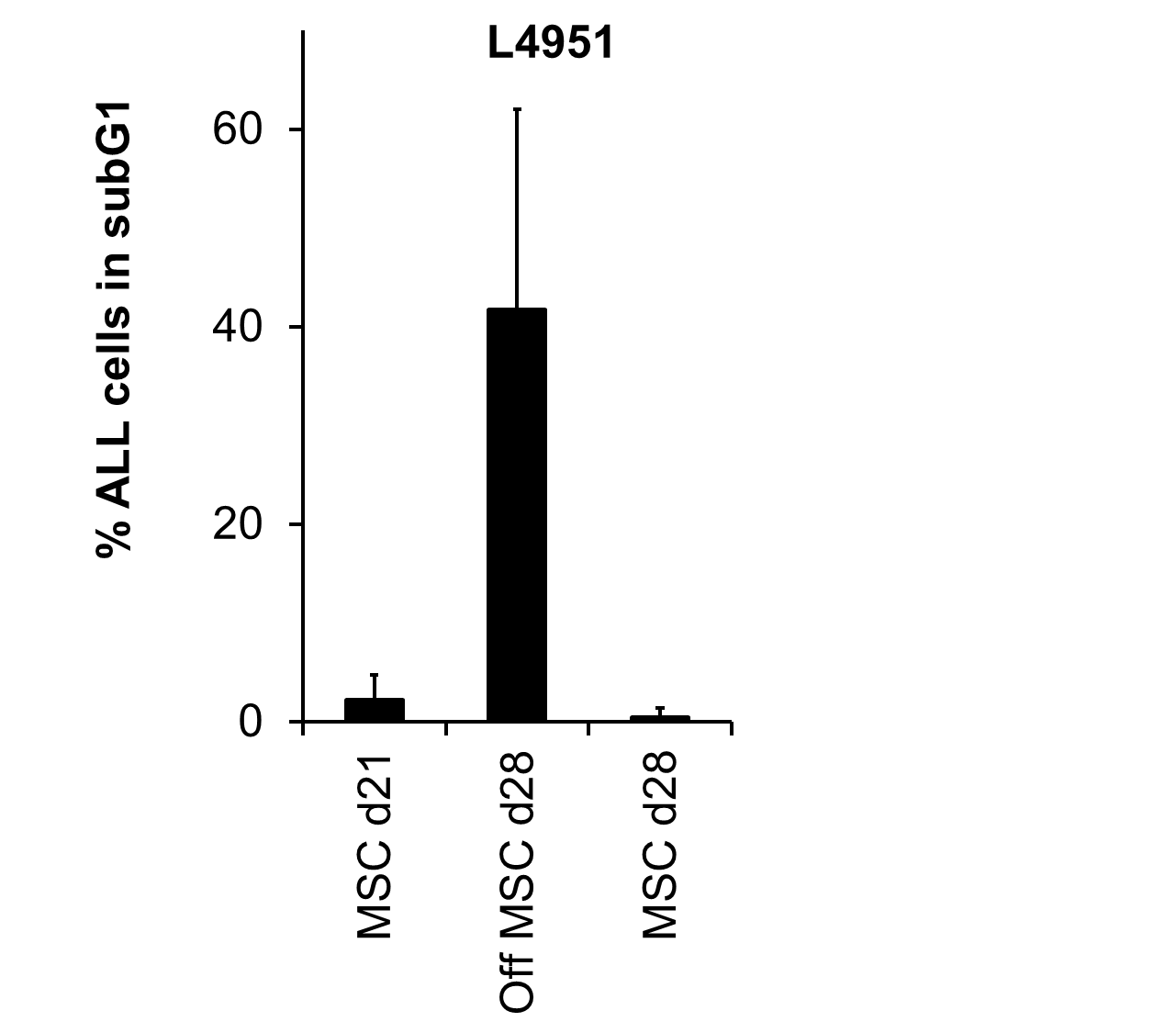

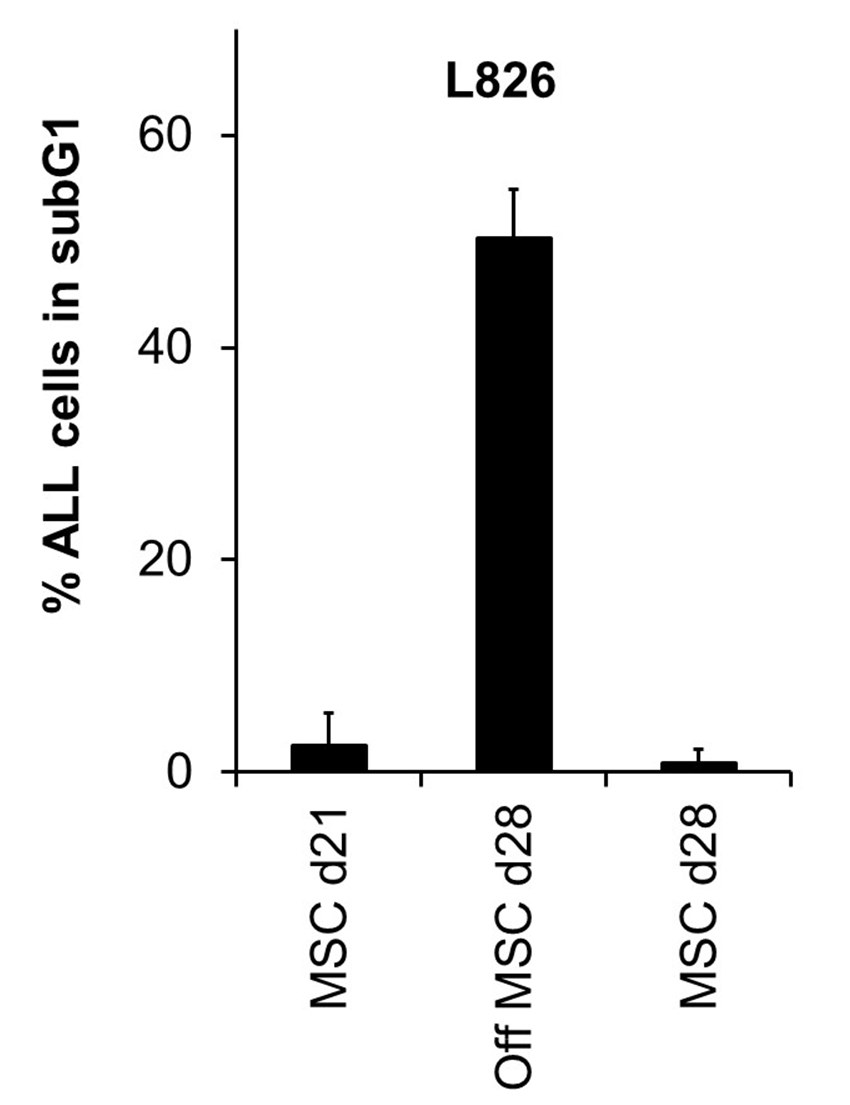


**a**

**B**

Figure S10: B-ALL cells retain feeder-dependence following long-term expansion on MSC. The graphs show the percentage of ALL cells in subG1 for the BCR/ABL- positive ALL patient L4951 (A) and the MLL/AF4-positive patient L826 on feeders at days 21 and 7 days after removal from the MSC feeder at day 28. N = 3 experiments; error bars, SD.


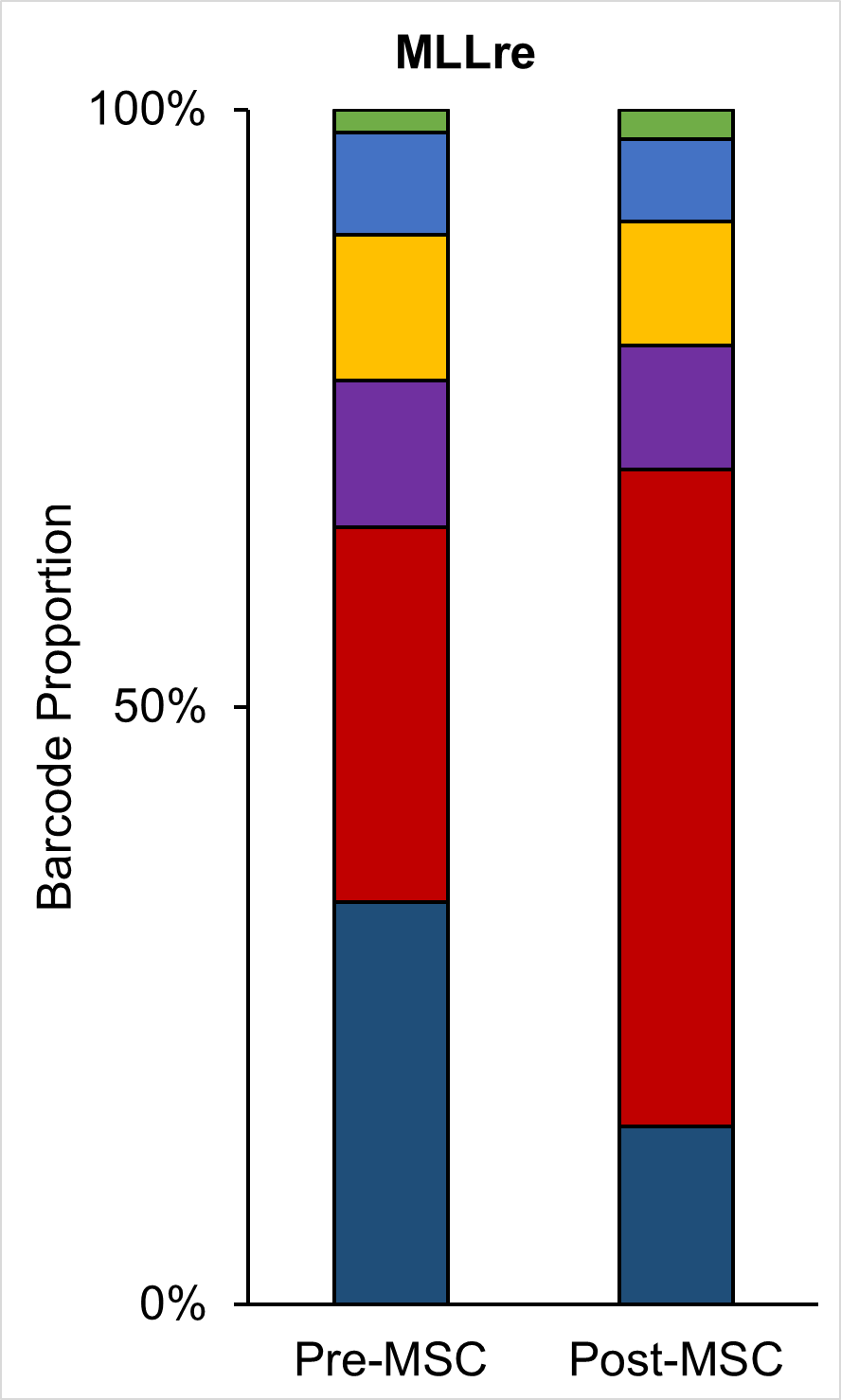


Figure S11. Clonal composition of a low complexity barcoded MLLre sample (baseline) and after 2-week expansion on MSCs. Each colour represents a distinct clone.


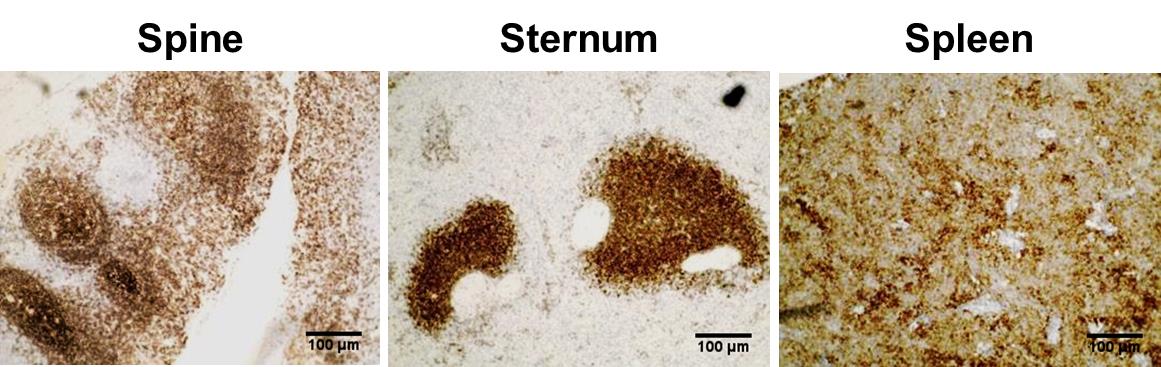


**A**

**B**

**C**

**D**


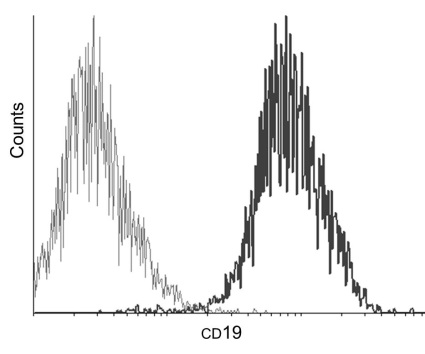

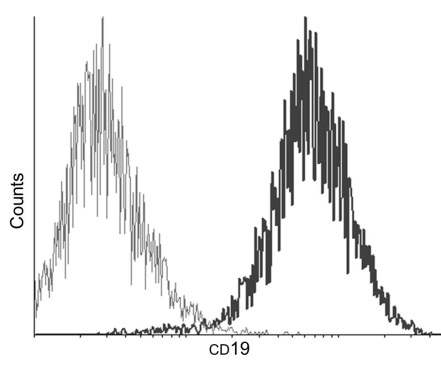

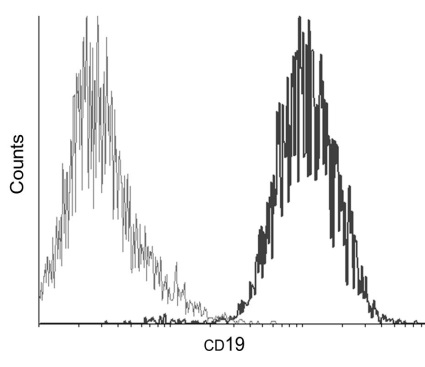


Figure S12: B-ALL cells engraft NSG mice after prolonged co-culture on MSCs. A, Histopathological analysis of engrafted organs confirm positive human CD19 expression in spine, sternum and spleen. B-D, FACS analysis of CD19 expression of ALL cells harvested from mouse calvaria (B), bone marrow (C) and spleen (D). Grey Isotype control; black, CD19-stained cells. N = 6 experiments

**Table S1: Clinical data for primary and primograft ALLs**

**Table S2: Antibodies**

**Supplemental Methods**

***Patient material***

Primary bone marrow and peripheral blood samples from children with B-lineage ALL (B-ALL) were collected as part of the initial diagnostic investigations. Primograft samples were derived by passaging individual primary patient samples through NSG mice. Samples were collected, stored and used following written informed consent from patients and/or legal guardians according to protocols approved by the corresponding review boards. Samples were retrieved from the Newcastle Haematology Biobank under generic biobank approval given by the NRES Committee North East - Newcastle & North Tyneside 1 (REC reference number 07/H0906/109+5).

***MSC derivation and culture***

Primary MSC cultures were established from 10 different individuals aged between 36 and 75 years, undergoing hip replacement surgeries for osteoarthritis. Samples were collected following appropriate consent and according to approval given by the Newcastle and North Tyneside 1 Research Ethics Committee (REC Reference Number: 09/H0906/72) and processed within 24 hours of surgery. Trabecular bone fragments were dissected out from the femoral head and processed over a 1.077 g/ml Lymphoprep™ density gradient medium (Stemcell technologies, Cambridge, UK). Mononuclear cells including putative MSC were visible as an opaque band at the Lymphoprep^TM^-marrow interphase. The mononuclear band was washed in MSC wash buffer (5mM EDTA/0.2% BSA/1% penicillin-streptomycin) and resuspended in low glucose DMEM (1000 mg/ml) (Sigma, UK) reconstituted with 20% fetal calf serum (GIBCO®, Life Technologies, Paisley, UK), 1% L-glutamine (Sigma, UK) and 1% penicillin-streptomycin (Sigma, UK). Healthy MSC adhere to plastic within 24 hours. At this point the cells were washed with MSC wash buffer to avoid contaminants and fresh media was added, with the addition of bFGF (GIBCO®, Life Technologies, Paisley, UK) at 8 ng/ml. MSC were used between passages 2-5 and within 40 days following surgery.

***Co-culture of ALL cells on MSC***

h-TERT MSC (Applied Biological materials, Inc., Canada) and MSC were seeded at a density of 10^4^ cells/cm^2^ in MSC media 48 hours prior to adding B-ALL. B-ALL cells were seeded onto MSC at a density of 2x10^6^ cells/ml (L4951, L578, L707-R, L722, L754, L826, L868, L880, 19578) and 10^6^ cells/ml (L707) in B-ALL media comprising SFEM II medium (Stemcell technologies, Cambridge, UK) supplemented with 20% fetal calf serum (GIBCO®, Life Technologies, Paisley, UK), 20 ng/ml recombinant IL3 (R&D Systems, Abingdon, UK) and 10 ng/ml recombinant IL7 (R&D Systems, Abingdon, UK). B-ALL cells were harvested every 7 days. Non-adherent cells present in supernatant medium and two washes with PBS were collected followed by trypsination and collection of the adherent cell fraction, which contains both MSCs and adhered ALL cells. After passing through a 15 µm filter (pluriSelect Life Science, Leipzig, Germany), ALL cells were separated from MSCs by magnetic cell separation using CD-19 microbeads (Miltenyi Biotec Ltd., Surrey, UK). Viable ALL cells were counted by trypan blue exclusion, re-suspended in fresh B-ALL media and seeded onto fresh MSC.

***Cell cycle analysis***

Live Hoechst 33342/Pyronin Y staining was performed as described previously . B-ALL cells (10^6^ cells) were resuspended in 1ml of 10 µg/ml of Hoechst 33342 (Sigma, UK) followed by incubation at 37˚C for 45 minutes. For G0 analysis, cells were further incubated with 100 µg/ml of Pyronin Y for an additional 15 minutes, followed by transfer onto ice and analysis on a FACS Canto II flow cytometer (BD Biosciences, Oxford, UK). G0/G1 populations were identified using FlowJo and Cyflogic v 1.2.1; cell cycle distribution was modelled using Mod Fit LT™. Gating strategies are shown in Figure S1.

***Flow cytometry***

B-ALL cells (5x10^5^ cells) were permeabilised in 0.1% Triton X-100 permeabilisation buffer (for intracellular staining with Nestin only) and incubated with saturating levels of monoclonal antibodies (Table S2) against the relevant antibodies for 20 minutes at room temperature in the dark [^3^](#_ENREF_3). Stained cells were washed twice with PBS and analysed in 500 µl PBS/0.2% BSA on a FACS Calibur flow cytometer (BD Biosciences, Oxford, UK). Data were analysed using softwares Cell Quest Pro v 6 and Cyflogic v 1.2.1. Gating strategies for separating ALL cells from MSCs are shown in Figure S2.

**Cell culture**

Cell lines REH and M210B4 were cultured at a density of 1 million cells/ml and 10,000 cells/cm^2^ respectively in RPMI-1640 (Sigma, UK) reconstituted with 10% foetal calf serum (GIBCO®, Life Technologies, Paisley, UK) and 1% L-glutamine (Sigma, UK). For the purposes of limiting dilution assay, the REH cells were cultured in 4% foetal calf serum at a density of 5000 cells/ml.

***Immunofluorescence***

Immunofluorescence staining was performed as described previously [^4^](#_ENREF_4). Cells were fixed in 4% paraformaldehyde and stained with relevant primary antibody (see supplementary table 2) overnight at 4˚C. Secondary antibodies used were Alexa Fluor® 594 Goat anti-mouse, Alexa Fluor® 488 Goat anti-rabbit, Alexa Fluor® 594 Goat anti-rabbit (all 1:500, Life technologies, Paisley, UK). The cells were mounted in Vectashield hardset antifade mounting medium (Vector laboratories, Peterborough, UK) with 4’,6-diamidino-2-phenylindole (DAPI) to counterstain the nuclei. Images were obtained using the Leica TCS SP2 UV AOBS MP point scanning confocal microscope (Leica microsystems, Cambridge, UK).

***Senescence associated β-galactosidase assay***

Following 4 weeks of co-culture on MSC the B-ALL cells were assessed for pH dependant β-galactosidase activity as per manufacturer’s protocol using the Senescence β-Galactosidase Staining Kit (Cell Signalling Technology, Hertfordshire, UK).

### Cellular Barcoding of ALL blasts

L707 cells were lentivirally marked with heritable genetic markers [^5-7^](#_ENREF_5). The thus barcoded L707 cells were expanded on MSC for six weeks with B-ALL replating on fresh MSC occurring every week. The barcoded regions in the thus expanded L707 – PDM were then amplified by means of PCR and sequenced using an Illumina MiSeq. Reads which contained the expected fixed sequences within the barcode and had Illumina quality scores above 20 were extracted using a customised python code.

***Drug treatment***

L707 and L707 – R cells were co-cultured on B-ALL for 48 hours at a B-ALL seeding density of 1 million cells/ml in B-ALL media. Dexamethasone was dissolved in methanol at up to stock concentrations of 25 mg/ml and ABT-199 was dissolved in DMSO at up to stock concentrations of 1mM. The cells were treated with Dexamethasone (Sigma, UK) at doses 0.01, 0.05, 0.5 and 1 µg/ml and ABT-199 (Selleck Chemicals, Munich, Germany) doses 0.08 µg/ml and 0.8 µg/ml. B-ALL cells were treated with the respective drugs for a period of six days. Clinically relevant dexamethasone plasma concentrations range between 0.01 µg/ml to 0.25 µg/ml over an eight hour period following drug administration in patients. Phase I dose escalation studies document ABT-199 plasma concentrations to range from 0.05 µg/ml to 1.0 µg/ml.

Drug combination indices were calculated using CalcuSyn Version 2.1[^8^](#_ENREF_8).

### Animal studies

Animal studies were conducted in accordance with UK Home Office regulations. B-ALL cells were lentivirally labelled as described previously to facilitate *in vivo* tracking . Between 10^4^ and 5x10^5^ cells of either primograft or primograft expanded on MSCs were transplanted into the right femur of NSG mice as described previously [^3^](#_ENREF_3). B-ALL engraftment was monitored by bioluminescent imaging following intraperitoneal injection of luciferin at a dose of 150 mg/kg (Promega, Southampton, UK) using an IVIS Spectrum (Caliper Life Sciences, MA, USA). Mice were killed following clinical evidence of leukaemia such as hind limb weakness, inactivity, hepatosplenomegaly or significant weight loss. Organ engraftment was examined by staining with human CD19.

**Supplemental References:**

1. Passegue E, Wagers AJ, Giuriato S, Anderson WC, Weissman IL. Global analysis of proliferation and cell cycle gene expression in the regulation of hematopoietic stem and progenitor cell fates. *The Journal of experimental medicine* 2005 Dec 5; **202**(11)**:** 1599-1611.

2. Santaguida M, Schepers K, King B, Sabnis AJ, Forsberg EC, Attema JL*, et al.* JunB protects against myeloid malignancies by limiting hematopoietic stem cell proliferation and differentiation without affecting self-renewal. *Cancer cell* 2009 Apr 7; **15**(4)**:** 341-352.

3. Rehe K, Wilson K, Bomken S, Williamson D, Irving J, den Boer ML*, et al.* Acute B lymphoblastic leukaemia-propagating cells are present at high frequency in diverse lymphoblast populations. *EMBO molecular medicine* 2013 Jan; **5**(1)**:** 38-51.

4. Moad M, Pal D, Hepburn AC, Williamson SC, Wilson L, Lako M*, et al.* A novel model of urinary tract differentiation, tissue regeneration, and disease: reprogramming human prostate and bladder cells into induced pluripotent stem cells. *European urology* 2013 Nov; **64**(5)**:** 753-761.

5. Elder AK, Heidenreich O, Vormoor HJ. Lentiviralmarking as a tool to investigate the clonal complexity and evolution of ALL. *Klin Padiatr* 2012 //27.04.2012; **224**(03)**:** A1.

6. Bomken S, Buechler L, Rehe K, Ponthan F, Elder A, Blair H*, et al.* Lentiviral marking of patient-derived acute lymphoblastic leukaemic cells allows in vivo tracking of disease progression. *Leukemia* 2013 Mar; **27**(3)**:** 718-721.

7. Gerrits A, Dykstra B, Kalmykowa OJ, Klauke K, Verovskaya E, Broekhuis MJ*, et al.* Cellular barcoding tool for clonal analysis in the hematopoietic system. *Blood* 2010 Apr 1; **115**(13)**:** 2610-2618.

8. Chou TC. Drug combination studies and their synergy quantification using the Chou-Talalay method. *Cancer research* 2010 Jan 15; **70**(2)**:** 440-446.

9. Scherr M, Elder A, Battmer K, Barzan D, Bomken S, Ricke-Hoch M*, et al.* Differential expression of miR-17~92 identifies BCL2 as a therapeutic target in BCR-ABL-positive B-lineage acute lymphoblastic leukemia. *Leukemia* 2014 Mar; **28**(3)**:** 554-565.
